# Secretome Analysis from the Ectomycorrhizal Ascomycete *Cenococcum geophilum*

**DOI:** 10.3389/fmicb.2018.00141

**Published:** 2018-02-13

**Authors:** Maíra de Freitas Pereira, Claire Veneault-Fourrey, Patrice Vion, Fréderic Guinet, Emmanuelle Morin, Kerrie W. Barry, Anna Lipzen, Vasanth Singan, Stephanie Pfister, Hyunsoo Na, Megan Kennedy, Simon Egli, Igor Grigoriev, Francis Martin, Annegret Kohler, Martina Peter

**Affiliations:** ^1^Institut National de la Recherche Agronomique, Unité Mixte de Recherche 1136 Interactions Arbres, Microorganismes, Laboratoire D'excellence Recherches Avancés sur la Biologie de l'Arbre et les Ecosystémes Forestiers, Centre Institut National de la Recherche Agronomique-Lorraine, Champenoux, France; ^2^Swiss Federal Research Institute WSL, Forest Dynamics, Birmensdorf, Switzerland; ^3^Université de Lorraine, Unité Mixte de Recherche 1136 Interactions Arbres-Microorganismes, Vandoeuvre les Nancy, France; ^4^United States Department of Energy Joint Genome Institute, Walnut Creek, CA, United States

**Keywords:** *Cenococcum geophilum*, small secreted proteins, ectomycorrhiza, symbiosis, interaction

## Abstract

*Cenococcum geophilum* is an ectomycorrhizal fungus with global distribution in numerous habitats and associates with a large range of host species including gymnosperm and angiosperm trees. Moreover, *C. geophilum* is the unique ectomycorrhizal species within the clade Dothideomycetes, the largest class of Ascomycetes containing predominantly saprotrophic and many devastating phytopathogenic fungi. Recent studies highlight that mycorrhizal fungi, as pathogenic ones, use effectors in form of Small Secreted Proteins (SSPs) as molecular keys to promote symbiosis. In order to better understand the biotic interaction of *C. geophilum* with its host plants, the goal of this work was to characterize mycorrhiza-induced small-secreted proteins (MiSSPs) that potentially play a role in the ectomycorrhiza formation and functioning of this ecologically very important species. We combined different approaches such as gene expression profiling, genome localization and conservation of MiSSP genes in different *C. geophilum* strains and closely related species as well as protein subcellular localization studies of potential targets of MiSSPs in interacting plants using in tobacco leaf cells. Gene expression analyses of *C. geophilum* interacting with *Pinus sylvestris* (pine) and *Populus tremula* × *Populus alba* (poplar) showed that similar sets of genes coding for secreted proteins were up-regulated and only few were specific to each host. Whereas pine induced more carbohydrate active enzymes (CAZymes), the interaction with poplar induced the expression of specific SSPs. We identified a set of 22 MiSSPs, which are located in both, gene-rich, repeat-poor or gene-sparse, repeat-rich regions of the *C. geophilum* genome, a genome showing a bipartite architecture as seen for some pathogens but not yet for an ectomycorrhizal fungus. Genome re-sequencing data of 15 *C. geophilum* strains and two close relatives *Glonium stellatum* and *Lepidopterella palustris* were used to study sequence conservation of MiSSP-encoding genes. The 22 MiSSPs showed a high presence-absence polymorphism among the studied *C. geophilum* strains suggesting an evolution through gene gain/gene loss. Finally, we showed that six CgMiSSPs target four distinct sub-cellular compartments such as endoplasmic reticulum, plasma membrane, cytosol and tonoplast. Overall, this work presents a comprehensive analysis of secreted proteins and MiSSPs in different genetic level of *C. geophilum* opening a valuable resource to future functional analysis.

## Introduction

Symbiotic plant–fungal interactions are predominant in worldwide soils and have important roles in the global colonization by land plants. In forest soils, the ectomycorrhizal (ECM) symbiosis is the dominant form of a mutualistic interaction between the fine roots of trees and fungal hyphae. This interaction allows the exchange of nutrients and water between partners and increases the disease resistance of host plants (Smith and Read, 2010). Approximately 20,000 ECM fungi from diverse fungal clades and about 6,000 tree species worldwide are able to form this association (van der Heijden et al., 2015; Martin et al., 2016). Although the ECM lifestyle evolved independently several times from ancestral saprotrophs (Hibbett et al., 2000; Kohler et al., 2015) the arisen symbiotic organ and mutualistic interaction is surprisingly similar each time. Recent genomic and transcriptomic studies indicate the convergent evolution of a symbiosis toolkit with two major features of the ECM lifestyle: a reduced number of plant cell wall degrading enzymes as compared to saprotrophic ancestors in the genome and in the transcriptome the accumulation of lineage-specific transcripts possibly involved in the biotic interaction (Kohler et al., 2015; Martin et al., 2016).

Ectomycorrhiza formation is a process controlled by different genetic and environment factors (Tagu et al., 2002; Smith and Read, 2010; Kohler et al., 2015; Martin et al., 2016). A molecular communication between fungi and plant is a prerequisite for establishment of a symbiotic interaction (Plett and Martin, 2011; Martin et al., 2016). In order to manipulate host defenses and enable colonization, the secretion of small proteins is a known mechanism of pathogenic fungal–host interactions. It has been observed in mycorrhizal interactions as well but their role is still poorly understood (Martin et al., 2008, 2016; Garcia et al., 2015; Plett and Martin, 2015). Fungal genome availability allowed comparative analyses across different lifestyles including saprotrophic, mycorrhizal, pathogenic and endophytic ones revealing that all fungal genomes encode for small-secreted proteins (SSPs), which are defined as proteins of <300 amino-acids containing a signal-peptide (Martin et al., 2008, 2010, 2016; Kohler et al., 2015; Pellegrin et al., 2015; Kamel et al., 2017). Despite some overlap among shared SSPs in ECM fungi and saprotrophic fungi based on sequence similarities, many genes encoding SSPs are orphan genes and are unique to each ECM species (Kohler et al., 2015; Pellegrin et al., 2015). To understand the role of SSPs in the mycorrhiza development, both gene expression studies as well as functional analyses are necessary. So far, only two SSPs, a Mycorrhizae induced Small Secreted Protein of 7Kda (MiSSP7) in *Laccaria bicolor*, an ECM fungus and the secreted protein 7 (SP7) in *Rhizophagus irregularis* (previously known as *Glomus intraradices*), an arbuscular mycorrhizal fungus have been functionally characterized, and in both cases, the secreted effector targeted to the host plants nucleus and reshuffled plant defense pathways (Kloppholz et al., 2011; Plett et al., 2011, 2014).

One of the most abundant ECM fungi is the ascomycete *Cenococcum geophilum* Fr. showing a worldwide distribution through numerous habitats, environments and geographic regions and associating with a large variety of host species including gymnosperms and angiosperms (Trappe, 1962; LoBuglio, 1999; Obase et al., 2017). *C. geophilum* forms characteristic black monopodial or dichotomous ectomycorrhizas with darkly pigmented, emanating hyphae, as well as resistance propagules known as sclerotia, but sexual structures have never been found (Trappe, 1962; LoBuglio, 1999; Obase et al., 2017). Although being a broadly distributed fungus, the biology of *C. geophilum* is poorly understood. Studies on the fine-scale diversity of *C. geophilum* populations revealed a high level of genetic polymorphism and this can help to explain the large amount of physiological and phenotypic differences reported among *C. geophilum* isolates from similar as well as diverse geographic regions (LoBuglio, 1999; Douhan et al., 2007; Obase et al., 2017). Likewise, the variability in genome size, ploidy level and gene polymorphism among *C. geophilum* isolates support the evidence of possible cryptic sexual recombination and speciation (Spatafora et al., 2012; Bourne et al., 2014). *C. geophilum* is the only ECM fungus belonging to the clade of Dothideomycetes, the largest class of Ascomycota with a high level of ecological diversity, including many devastating plant pathogens and saprotrophs (LoBuglio, 1999; Ohm et al., 2010).

The recent genome sequencing of a *C. geophilum* strain revealed a large size of 178 Mbp and is predicted to encode for 14,748 gene models (Peter et al., 2016). Transcript profiling of *C. geophilum* genes expressed in pine ectomycorrhizal root tips revealed the upregulation of genes encoding membrane transporters, including aquaporin water channels and sugar transporters in symbiosis. Also, MiSSPs were highly induced or even specifically expressed in symbiotic tissues as seen for other ECM fungi (Kohler et al., 2015; Peter et al., 2016). Furthermore, comparative genome analysis of *C. geophilum* with sequenced Dothideomycetes and a set of other fungi revealed that eight of the symbiosis-induced (>5 fold) SSPs are unique to *Cenococcum* and might play an important role in the fungal-plant interaction as seen for effector genes (Peter et al., 2016). One of the most striking features of the *C. geophilum* genome is its massively increased size compared to other sequenced Dothideomycetes (Peter et al., 2016). The 3–4 times larger genome of this ECM species is explained by the proliferation of transposable elements (TE), which make up 75% of the genome (Peter et al., 2016). Increased genome sizes due to TE bursts have been observed for other mycorrhizal fungi such as *Tuber melanosporum* and *Rhizophagus irregularis* (Kohler and Martin, 2016), but also for many biotrophic plant pathogens (Raffaele and Kamoun, 2012; Stukenbrock and Croll, 2014). In plant pathogens, these TEs are often not randomly spread over the genome but cluster in repeat-rich chromosomal segments that evolve at accelerated rates than the rest of the genome due to diverse mechanisms such as TE-activity and TE silencing machineries (Raffaele and Kamoun, 2012). Also, genes implicated in virulence and host adaptation such as effector genes tend to localize in repeat-rich, faster evolving regions (Raffaele and Kamoun, 2012). Even within species, substantial presence/absence polymorphisms have been observed for such genes in proximity of TEs for a plant pathogen, being a source of variation and driving local adaptation (Hartmann and Croll, 2017). Such a two-speed genome has convergently evolved in plant pathogenic fungi in independent lineages such as the oomycetes and the Dothideomycetes (Dong et al., 2015) but has not been observed for mycorrhizal fungi so far.

In order to better understand the biotic interaction of *C. geophilum* with its host plants, the goal of this work was to analyze whether *C. geophilum* is secreting MiSSPs as mean of communication with its host plants and narrow down the repertoire of candidate effectors for further demonstration. The specific objectives were (i) to assess the regulation of the *C. geophilum* secretome in ectomycorrhizal root tips formed with two different host plants, the gymnosperm *Pinus sylvestris* (pine) and the angiosperm *Populus tremula* × *Populus alba-*INRA clone 717-1-B4 (poplar) through transcriptomic analyses (ii) to identify candidate symbiosis effector genes and study their genomic localization, (iii) to study presence-absence polymorphism in candidate effectors by analyzing 15 re-sequenced *C. geophilum* strains and two closely related Dothideomycetes genomes to elucidate their evolution and conservation and (iv) to obtain a first glimpse of the possible role as effectors for a selection of MiSSPs by studying their potential target within the host plant cell through sub-cellular localization experiments in *Nicotiana benthamiana* leaf cells.

## Materials and methods

### Microorganisms growth condition

*Cenococcum geophilum* isolates originating from different sites (Supplementary Table S1) were kept in Petri dishes (100 × 20 mm) containing *Cenococcum* medium, a modified MMN medium containing casein (Trappe, 1962), at 25°C and transferred to new culture medium every 20 days. *Escherichia coli* (subcloning efficiency DH5a competent cells; Invitrogen, Carlsbad, CA, U.S.A.) and *Agrobacterium tumefaciens* (electrocompetent strain GV3101) were conserved at −80°C and they were grown in LB and YEPD medium at 37 and 28°C, respectively.

### Plant growth condition and ectomycorrhiza formation

*In vitro* interaction systems were established between *C. geophilum* isolate 1.058, of which the genome is available (http://genome.jgi.doe.gov/Cenge3/Cenge3.info.html) and Scots pine (*Pinus sylvestris*) or hybrid poplar (*Populus tremula* × *Populus alba*; INRA clone 717-1-B4) respectively. Pine seeds [*P. sylvestris* provenance VS/Leuk (31/10) WSL] were superficially disinfected in a laminar flux hood by immersion in H_2_O_2_ for 30 min, followed by three rinses with sterile distilled water. The seeds were germinated in modified MMN medium described by Brun et al. (1995), with low nitrogen and phosphorus during seven days for observation of contamination. After seed germination, the plants were transferred to Petri dishes containing modified MMN and covered with a cellophane membrane (135 mm). Approximately ten agar disks containing fungal mycelium of *C. geophilum* 1.58 were placed in the vicinity of the roots. The dishes were incubated in a growth chamber at 25°C with 16 h light/day for 90 days (Supplementary Figures S1A,B,E).

The hybrid poplar (*Populus tremula* × *Populus alba*; INRA clone 717-1-B4) was micropropagated *in vitro* in Murashige and Skoog (MS) medium (Murashige and Skoog, 1962), with hormone supplements to synchronize rhizogenesis as described by Felten et al. (2009). In parallel, the MNM medium with low phosphorus and nitrogen (Brun et al., 1995) was covered with cellophane membranes and inoculated with 10–12 agar disks containing fungal mycelium at 25°C for 20 days. Following this, two hybrid poplar plants per dish were transferred onto the fungal mycelium. The Petri dishes were incubated in a growth chamber at 25°C with 16 h light/day for 60 days (Supplementary Figures S1C–E).

For both experiments, pure cultures of *C. geophilum*, pine and hybrid poplar grown in identical conditions were used as experimental controls, and the assays were conducted in minimum of three replicates. After the indicated period of time, Petri dishes were opened and the different tissues were collected for RNA analyses as follows: Single mycorrhizal root tips were collected in a 1.5 ml tube and immediately frozen in liquid nitrogen. Extramatrical mycelium surrounding roots and emanating from pine ECMs was scratched from the cellophane using a scalpel and if present, sclerotia formed in these dishes were separately collected and also immediately frozen in liquid nitrogen. For pure culture controls, free-living mycelium or fine root tips, respectively, were collected at the same time and manner as indicated for synthesis Petri dishes.

### RNA extraction and illumina sequencing and data analysis

Total RNA from mycorrhizal roots, sclerotia, extramatrical mycelium, fungal, and plant controls were extracted with the RNeasy Plant Mini kit (Qiagen, Courtaboeuf, France), including a DNase I (Qiagen) treatment, according to the manufacturer's instructions to eliminate traces of genomic DNA. Assays for the quantification and integrity check were conducted using an Experion Automated Electrophoresis Station (Bio-Rad, Hercules, CA, USA) or Agilent 2100 Bioanalyzer system (Agilent, Santa Clara, CA, USA).

Preparation of libraries and 2 × 150 bp Illumina HiSeq2000/2500 mRNA sequencing (RNA-Seq) was performed by the Joint Genome Institute (JGI) facilities. Raw reads were filtered and trimmed using the JGI QC pipeline (see Supplementary Table S2). Using BBDuk, raw reads were evaluated for artifact sequence by kmer matching (kmer = 25), allowing 1 mismatch and detected artifact was trimmed from the 3′ end of the reads. RNA spike-in reads, PhiX reads and reads containing any Ns were removed. Quality trimming was performed using the phred trimming method set at Q6. Finally, following trimming, reads under the length threshold were removed (minimum length 25 bases or 1/3 of the original read length—whichever is longer). Filtered reads from each library were aligned to *C. geophilum* v 2.0 reference transcripts available at the JGI database (http://genome.jgi.doe.gov/Cenge3/Cenge3.info.html). FeatureCounts was used to generate the raw gene counts and only primary hits assigned to the reverse strand were included in the raw gene counts (Liao et al., 2014). Raw gene counts were used to evaluate the level of correlation between biological replicates using Pearson's correlation and determine which replicates would be used in the DGE analysis. FPKM (Fragment Per Kilobase of exon model per Million mapped reads) and TPM (transcripts per million) normalized gene counts were also provided. DESeq2 (version 1.10.0), including an independent filtering procedure by default, was used to determine which genes were differentially expressed between pairs of conditions (Love et al., 2014). The parameters used to call a gene differentially expressed between conditions were fold change > log1 and FDR *p* < 0.05. A gene with a FPKM > 1 was considered as expressed. The complete RNA-Seq data was submitted to GEO (GSE108831 and GSE108866). For selecting MiSSPs as well as for comparisons among different synthesis systems, we added RNA-Seq data of a semi-sterile greenhouse trial growing *P. sylvestris* with *C. geophilum* 1.58 in pots (Peter et al., 2016; GEO Accession GSE83909). Here, pine trees were pre-grown for 2 months in pots containing a 1:2 double-autoclaved mixture of quartz sand and sieved forest topsoil before being inoculated by *C. geophilum* 1.58 mycelia and grown for another 3 months before harvesting ECMs. As pure culture fungal control, 2-months-old mycelium grown as indicated above on agar Petri dishes was used (Peter et al., 2016).

### Genome architecture and gene density analysis

Genomic distances between two genes and genome architecture heatmaps were generated according to Saunders et al. (2014). These results were binned according to log (length) and plotted as a 2-dimensional heatmap using Excel. Plotting the abundance of genes according to their 5′ and 3′ flanking intergenic lengths indicate local gene density (**Figure 3**). In *C. geophilum* genome, we defined two contrasting regions: one gene-dense repeat-poor (GDR) containing a high number of genes (gene-dense) combined with short 3′ and 5′ flanking regions indicating a low level of repeats (repeat-poor), whereas the gene-sparse repeat-rich (GSR) region is characterized by a low number of genes displaying long 5′ and/or 3′ flanking regions. We also represent, according to local gene density, the distribution of gene expression induction in ECM root tips compared to free-living mycelium (log2 fold change) or their level of expression (fpkm values).

### DNA extraction, genome re-sequencing of *Cenococcum geophilum* isolates and presence–absence analyses of selected SSPs

To study the presence/absence polymorphism of selected SSPs, data of 15 recently re-sequenced strains of *C. geophilum* was used. The 15 strains originated from diverse locations in Switzerland, France, Poland and Finland (Supplementary Table S1). For genomic DNA sequencing, mycelia were grown in liquid culture containing *Cenococcum* medium for 3–4 weeks after which they were harvested, pulverized in liquid nitrogen and stored at −80°C until processing. DNA was extracted using the PowerMax Soil DNA isolation kit (MOBIO/QIAGEN CA, USA) according to the manufacturer's instructions and using around 2 g of mycelia. Library construction and sequencing was performed at the Joint Genome Institute (JGI) using Illumina HiSeq 2500 and 2 × 100 bp read length sequencing in two different lanes. Between 26 and 48 million raw reads were generated corresponding to a 15–27x coverage. CLC genomic workbench 10 was used to de novo assembly the 15 genomes with the following parameters: Mapping mode: map reads to contigs; minimum contig lenght: 500; Mismatch cost = 2; Insertion cost = 3; Deletion cost = 2; length fraction = 1.0; Similarity fraction = 0.9. A summary is given in Supplementary Table S3 and sequence contigs for the different strains and for all MiSSPs studied are compiled in Supplementary File 1 (http://mycor.nancy.inra.fr/IMGC/CenococcumGenome/download/Supplementary_data_1.fa.gz).

Screening for presence–absence polymorphism of the 22 selected *C. geophilum* MiSSPs and 22 Core eukaryotic genes–CEG (Supplementary Table S4) in the 15 re-sequenced strains was done by conducting a BLASTN search against the *de novo* assemblies and the reference genome 1.58 (https://genome.jgi.doe.gov/Cenge3/Cenge3.home.html). Further, the genome data of the closest related species, *Glonium stellatum* (https://genome.jgi.doe.gov/Glost2/Glost2.info.html) and *Lepidopterella palustris* (https://genome.jgi.doe.gov/Leppa1/Leppa1.home.html) was used to compare *C. geophilum* SSP sequences for polymorphism (Peter et al., 2016). A gene was considered as affected if the deletion event was overlapping >90% of the gene. To check presence–absence polymorphism in gene duplications, manual alignments was done using the INRA Multalin interface (Corpet, 1988). The presence of a *C. geophilum* MiSSP in the respective *de novo* assembly contigs was defined as the lowest e-value accession (E-value) combined with the greatest HSP length (number of nucleotides in the reference genome–Cg1.58).

The variability in presence–absence patterns of the 22 selected MiSSP genes among *C. geophilum* isolates was examined with principal coordinate analyses (PCO) using the Jaccard similarity index. Variation explained in these patterns by phylogenetic clade (3 levels), country (4 levels) and forest type (4 levels) of isolate origin were assessed using the PERMANOVA routine (Anderson, 2001) implemented in the software Primer7 using 9,999 unrestricted permutations of raw data as well as by Monte Carlo tests (Clarke and Gorley, 2015). Phylogenetic analysis was performed using the online software phylogeny.fr from concatenated sequences of *C. geophilum* GAPDH and ITS using default parameters (Dereeper et al., 2008). In short, MUSCLE was used to align sequences and Gblocks for curation. Phylogeny was performed using a maximum likelihood algorithm using PhyML and branch confidence indices were calculated based on an approximate likelihood ratio test. ITS and GAPDH sequences are given in Supplementary Table S5.

### Validation of SSP gene presence–absence in different *Cenococcum geophilum* isolates by PCR

We validated gene presence–absence polymorphism for some selected *C. geophilum* MiSSPs using direct amplification of target genes including upstream and downstream regions. The primers were designed using Primer 3.0 (Untergasser et al., 2012) from a conserved flanking sequences of each gene (Supplementary Table S6). PCR reactions were performed with OneTaq® DNA Polymerases according to the manufacturer's instructions (New England Biolabs, Mass, USA) and amplicons run on 1% agarose gels. Each PCR reaction was purified with QIAquick PCR Purification Kit (Qiagen, Courtaboeuf, France) and the PCR product verified by sequencing (Eurofins, Ebersberg, Germany).

### Cloning procedures and plasmids used for localization experiments

The open reading frame (ORF) coding the mature form (i.e., without the signal peptide) of 22 *C. geophilum* selected MiSSPs were synthetized by GeneCust Europe (Ellange, Luxembourg). The vectors were designed with *att* sites accomplish to gene sequence to be compatible with PCR Cloning System with Gateway® Technology. The entry clone (*C. geophilum* MiSSP vectors) was utilize in LR recombination reaction with pB7WGF2 (C-terminal fusion with GFP) destination vector to create an expression clone (Karimi et al., 2002). The vectors were amplified in *E. coli* (DH5a competent cells; Invitrogen, Carlsbad, CA, USA). Sequences of DNA fragments inserted in vectors obtained by PCR were verified by sequencing (Eurofins genomics, Ebersberg, Germany) before to clone in *A. tumefaciens* (electrocompetent strain GV3101). For colocalization studies, we used a set of markers fused to mCherry protein developed by Nelson et al. (2007).

### Transient protein expression in *Nicotiana benthamiana* leaf cells

*N. benthamiana* plants were grown in phytotron at 22°C under 16-h day and 8-h night conditions. *A. tumefaciens* GV3101 was used to deliver T-DNA constructs into leaf cells of 4–6 weeks-old *N. benthamiana* plants, following the agroinfiltration method previously described (Win et al., 2011). Overnight-grown bacterial cultures were resuspend into 10 ml of infiltration buffer (10 mM MgCl2, 10 mM MES, pH 5.6, 200 μM acetosyringone), optical density at 600 nm (OD600) adjusted at 0.1. Bacteria were incubated at 28°C during 2 h under 50 rpm. For all co-transformations, *A. tumefaciens* strains were mixed in a 1:1 ratio in infiltration buffer to a final OD 600 of 0.2. The leaves were collected 2 days after infiltration for further protein isolation or microscopy analysis.

### Live-cell imaging by laser-scanning confocal microscopy

Small pieces of leaves were mounted in Perfluorodecalin 95% (Sigma-Aldrich, Saint Louis, MO, USA) and water between a slide and a coverslip and were immediately observed. Live-cell imaging was performed with a Zeiss LSM780, confocal microscope system, using 10× (air) and 40× (water immersion) objectives. The GFP was excited at 488 nm, whereas the mCherry was excited at 561 nm. Specific emission signals corresponding to the GFP and the mCherry were collected between 505–525 and 580–620 nm, respectively. Each construct gave a similar localization pattern across at least three independent observations. After observation, leaves were frozen in liquid nitrogen and were conserved at −80° C for further use.

### Total protein isolation and immunoblotting

*N. benthamiana* leaves were harvested 2 days after infiltration, were frozen in liquid nitrogen, and were ground into powder with mortar and pestle. Total protein extraction was performed by reducing and denaturing proteins from the leaf powder 10 min at 95°C in Laemmli buffer (0.5 M Tris-HCl, pH 6.8, 10 mM dithiothreitol [DTT], 2% SDS, 20% glycerol) in order to avoid *in vitro* nonspecific degradation of the fusion proteins. Proteins were separated by 15–20% SDS-PAGE (Mini-PROTEAN® TGX™ Gels) and transferred onto a nitrocellulose membrane using Trans-Blot Turbo Transfer System (Bio-Rad, CA, USA). Transfert efficiency was assessed by Red Ponceau staining. GFP detection was performed in a single step using a GFP (B2): sc-9996 horseradish peroxidase (HRP)-conjugated antibody (Santa Cruz Biotechnology, Santa Cruz, CA, USA). Protein bands on immunoblots were detected using Clarity ECL Western Blot Substrate (Bio-Rad, CA, USA) using the manufacturer's protocol.

## Results

### Host-dependent gene expression changes of *Cenococcum geophilum* secreted protein-encoding genes

The *C. geophilum* genome contains a total of 595 predicted secreted proteins (SP) including 227 Small Secreted Proteins (SSPs, <300 aa), 120 Carbohydrate-Active Enzymes (CAZymes), 13 lipases, 27 proteases, and 208 other SPs (Peter et al., 2016).

To study host dependent changes in the gene expression of secreted proteins we performed RNA-Seq analyses on *C. geophilum* ECM roots from *P. sylvestris* and *P. tremula x alba* and *C. geophilum* free living mycelium grown in *in vitro* systems. We complemented the analysis with samples from extramatrical mycelium of pine ECM and sclerotia.

**Figure 1:**
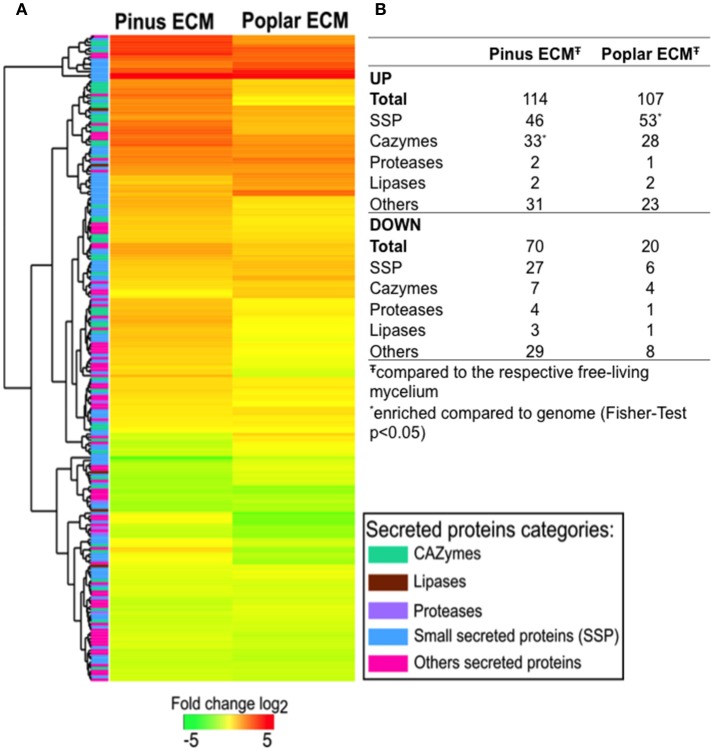
Changes in *C. geophilum* gene expression in pine and poplar ECM compared to free-living mycelium. Secretome transcripts significantly regulated (log2 fold > 1; FDR *p* < 0.05) in minimum one of the mycorrhizal systems to free-living mycelium were included in the analysis. **(A)** Fold change of gene expression was calculated for pine and poplar ECMs compared to the mean free-living mycelium expression value. Log2 transformed data were subjected to R Package Heatmap2 for clustering. Regulation levels range from pale to saturated colors (red for induction; green for repression). A column on the left side indicates by color the class of the secreted protein predicted for each gene. **(B)** Number of transcripts regulated in pine and poplar ECMs by secreted protein class. ^*^SP class enriched compared to the number present in the genome (Fisher-test for enrichment *p* < 0.05). Data are provided in Supplementary Table S7.

**Figure 2:**
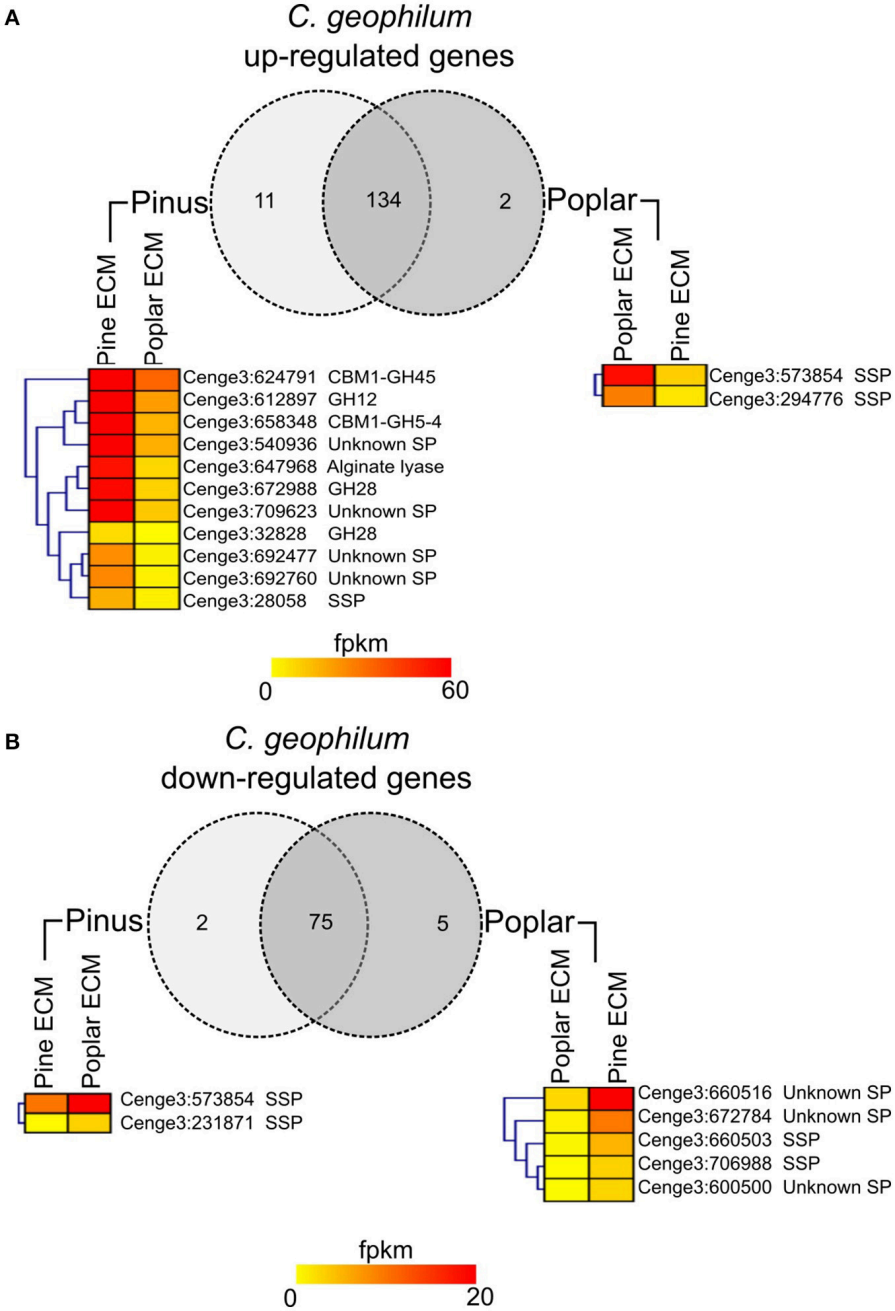
Host specific secretome of *C. geophilum*. Transcripts up-regulated **(A)** or down-regulated **(B)** in pine and/or poplar ECMs compared to free-living mycelium and their expression values (fpkm) in mycorrhizal tissue. Genes coding for secreted proteins were considered as used in both interactions if the expression difference was <5-fold. The expression was considered as more specific for one host tree if the expression differences was >5-fold. The expression of genes showing a more then 5-fold difference is shown in heatmaps for pine and poplar ECM. Note that the expression in some cases is <1 fpkm but never zero. Note that eight transcripts that were up-regulated in one and down-regulated in the other ECM were counted two times.

The majority of SPs were expressed in all tissues (88–93%). For 30 (5%) of them, no transcripts were detected in any of the conditions studied. The expression of 221 transcripts was significantly regulated in ECM root tips as compared to free-living mycelium in the *in vitro* systems (FC > log1, FDR *p* < 0.05; Figure 1; Supplementary Table S7). In interaction with pine roots, 114 SSPs were up- and 70 down-regulated, while in contact with poplar roots 107 SSPs were up- and only 20 down-regulated compared to control free-living mycelium (Figure 1B). The majority of genes were similarly regulated in the interaction with both host trees (Figure 1A). Among the most highly up-regulated transcripts in both interactions were SSPs (e.g., Cenge3:660401, Cenge3:693798, Cenge3:698167), but also secreted CAZymes (GH131, CBM1-GH45, CBM18-CE4-CBM18) (Supplementary Table S7). Interestingly, the up-regulated transcripts of pine ECM were significantly enriched in CAZymes, whereas for poplar ECM, they were enriched in SSPs (Figure 1B). We further analyzed the host-dependent expression levels of these 221 SPs in ECM of the different hosts. If the expression values varied less then five times between the two hosts, we considered a transcript as used in both interactions; with a more then five-fold difference, the transcript was considered as more important for either of the two host interactions (Supplementary Table S8). The majority of the genes (134/147 up-regulated, 76/83 down-regulated) were similarly expressed in ECM root tips of both host plants (Figure 2). None of the SPs was specific for one interaction; that is, showing no expression (value 0) when interacting with the other host tree. However, significantly higher expression was observed for two SSPs in interaction with poplar (Cenge3:573854; Cenge3:294776) and for one SSP and several CAZymes in interaction with pine roots (Cenge3:28058; CBM1-GH45, GH12, CBM1-GH5-4, GH28, GT90) (Supplementary Table S7). Interestingly, when comparing the different synthesis systems used, i.e., *in vitro* agar Petri dishes for pine and poplar and a semi-sterile pot system for pine (Peter et al., 2016), it seems that the system had a more pronounced impact on gene expression changes in interactions than had the host identity. Clearly more *C. geophilum* genes were commonly up-regulated in ECM of pine and poplar from the *in vitro* system (41 genes) as compared to commonly up-regulated genes in pine ECM using the different systems (6 genes; Supplementary Figure S2).

### Selecting mycorrhiza induced small secreted proteins (MiSSPs) for further characterization

We further focused on *C. geophilum* MiSSPs in order to identify candidate effector proteins for the interaction between *C. geophilum* and its host trees. A set of 22 MiSSPs, induced (>2.5 fold) in the interaction between *C. geophilum* and *P. sylvestris* under semi-sterile greenhouse conditions was selected from Peter et al. (2016) (Table 1). The predicted protein size of these MiSSPs ranged from 58 to 275 aa, containing no (e.g., Cenge3:664950 and Cenge3:679266) to 10.61% of cysteine residues (Cenge3:666290). Only six MiSSP sequences contain known domains or sequence homology to known proteins such as the SnoaL domain (PF12680, PF13577; Cenge3: 677330), the cupin domain (PF00190, PF07883; Cenge3:552209), the Ubiquitin 3 binding protein But2 C-terminal domain (PF09792; Cenge3:677232 and Cenge3:658610) or the “secreted in xylem 1” (Six1) protein of *Fusarium oxysporum* (Cenge3:698167; Rep et al., 2004). Eight MiSSPs were specific to *C. geophilum* while the others share sequence similarity with genes from other Dothideomycetes fungi (Table 1). Three pair of duplications were present within the selected MiSSPs showing sequence similarities from 77–95 to 72–93% for nucleotide and protein sequences, respectively (Supplementary Figure S3). Expression studies showed that some of these MiSSPs were also up-regulated in sclerotia formed in *in vitro* synthesis dishes and in extramatrical mycelium emanating from the ECM root tips (Table 1).

**Table 1:** List of 22 MiSSPs candidates selected of *Cenococcum geophilum* and *Pinus sylvestris* ectomycorrhiza.

						**Transcript evidence (FC log2)**				
**Protein ID**	**INTERPRO/putative function-best hit**	**Size (aa)**	**SP length**	**Cystein %**	**Presence in others Dothideomycetes fungi**^†^	**Semi-steril synthesis**	***In vitro*** **synthesis**			
						***P. sylvestris*** **ECM**	***P. sylvestris*** **ECM**	***P. tremula** × **P. alba*** **ECM**	**Extramatricial mycelium**	**Sclerotia**
28058	–	209	20	8.17	Yes	2.49	−0.92	2.5		
331593	_	81	21	6.25	No	1.42	−0.02	−0.7		
552209	Cupin domain, manganese ion binding/spherulin-like	275	20	1.46	Yes	4.41	2.1	0.87		
634429	Protein of unknown function DUF4237	224	18	1.79	Yes	2.43	3.6	3.6	x	x
636312	Duplication of Cenge3:660403	249	19	3.23	Yes	1.93	0.46	0.74		
658610	Ubiquitin 3 binding protein But2, C-terminal	186	19	1.08	Yes	3.55	−0.25	−0.82		x
659287	–	136	20	7.41	Yes	3.66	1.2	0.6		
659858	Duplication of Cenge3:660401	58	19	3.51	No	4.3	0.95	1.2		
660401	Duplication of Cenge3:659858	58	19	3.51	No	8.08	9.4	5.9		
660403	Duplication of Cenge3:636312	249	19	3.23	Yes	6.53	0.68	−0.074		
661585	_	194	22	4.66	Yes	5.4	3.5	2.3		x
664950	_	72	19	0	No	1.21	0.21	−0.39		
666290	–	180	22	10.61	Yes	2.95	2	3		x
667330	NTF2-like domain, Polyketide cyclase SnoaL-like domain	172	19	0.58	Yes	3.75	3.8	2.9		x
668273	–	204	19	0.99	Yes	3.53	2.7	3.4		x
670497	_	199	17	5.05	Yes	2.89	−0.041	0.15		
677232	Ubiquitin 3 binding protein But2, C-terminal	204	18	1.48	Yes	7.53	−6.2	−1.6		
679266	Duplication of Cenge3:693798	131	20	0	No	5.94	2.5	4.9		x
680403	_	135	20	1.49	No	7.88	1.5	5.4		x
693798	Duplication of Cenge3:679266	239	21	0.84	No	7.18	8.4	6.5		x
698167	*Fusarium* secreted in xylem protein 1	259	23	3.88	No	8.21	8.1	6.9		x
723230	–	96	23	2.11	Yes	2.52	0.3	0.26		

† *Presence in other Dothideomycetes of which genome sequences are available (https://genome.jgi.doe.gov/dothideomycetes/dothideomycetes.info.html) determinated by Blastp analysis*.

*FC, Fold change; SiP, Signal Peptide; ECM, ectomycorrhizal roots. GDR, Gene dense region; GSR, Gene sparse region*.

### Genome of *Cenococcum geophilum* displays a bipartite architecture with MiSSPs present in both regions

**Figure 3:**
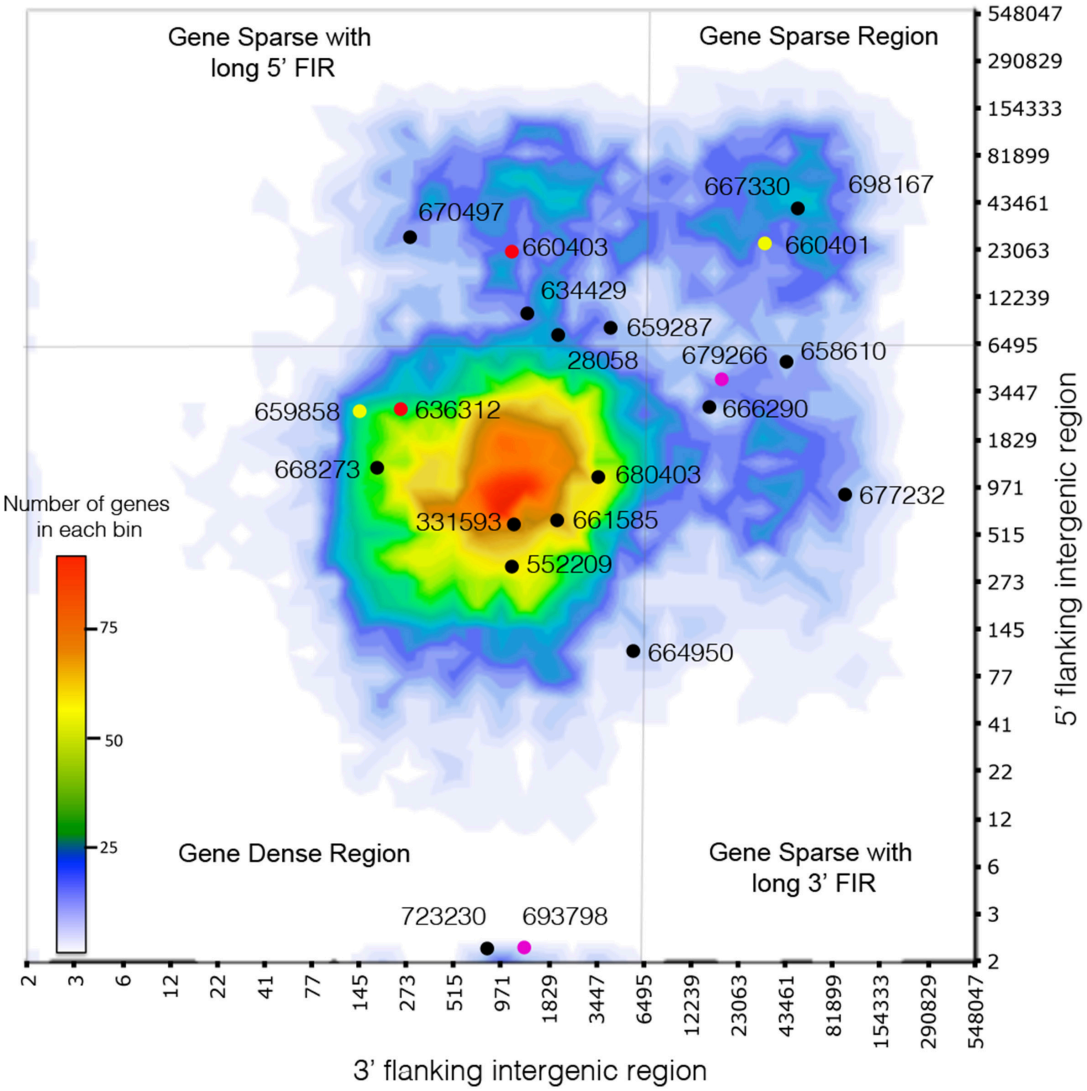
*Cenococcum geophilum* genome architecture. Distribution of distances of the closest repetitive genomic element on 5′ and 3′ sides for all genes (heatmap) and the genes encoding the 22 *C. geophilum* MiSSPs. Genes represented with the same color correspond to gene duplications. Gray lines delineate 5′ and 3′ intergenic flanking region (FIR) at 6495 bp to highlight long 5′ and 3′ intergenic flanking region and the gene-sparse repeat-rich regions (GSR) vs. the gene-dense repeat-poor region (GDR).

Due to richness in transposable elements found in the *C. geophilum* genome, we measured for each gene the distance to the neighboring genes at both 5′ and 3′ end. This method is used as a proxy to detect repeat-rich regions, assuming that the larger the intergenic region is the more repetitive sequences are present (Raffaele et al., 2010b). *C. geophilum* genome displayed two types of regions: repeat-rich, gene-sparse regions (GSR) and repeat-poor, gene-dense (GDR) regions, with a cut-off for 5′ and/or 3′ intergenic region length at >6,495 bp (Figure 3). This indicates a “two-speed” genome for *C. geophilum* as seen for some pathogenic fungi. In order to test whether gene position and environment could impact the *in planta* gene regulation, we measured the distribution of all *C. geophilum* genes for their expression induction and repression in ectomycorrhizal root tips compared to free-living mycelium according to local gene density. We observed that *in planta* regulated (either induced or repressed) genes are scattered all over the genome independently of the type of region. Neither did the host (pine vs poplar) nor the environmental condition (greenhouse vs. *in vitro*) influence this observation (Supplementary Figures S4A–C). Furthermore, gene location had an impact on the median level of gene expression (rpkm). Genes located in GSR (repeat-rich) tended to be expressed at a lower level than genes located in GDR (repeat-sparse) (Supplementary Figure S4D). This suggests an impact of repeats on the gene expression level.

One third (34%) of the genes encoding for the predicted *C. geophilum* secretome were located in GSR, which parallels the proportion found for the full-predicted proteome (33%; Supplementary Figure S5). Within the secretome, the categories “SSPs” and “other SPs” tended to show more members in GSR (37%) compared to the secreted CAZymes, lipases or proteases (28, 23, and 33%, respectively; Supplementary Figure S5) but no significant enrichment was observed. Again, we did not notice a difference in *in planta* gene regulation whether secretome-encoding genes were located in GDR or GSR (data not shown).

The 22 *C. geophilum* MiSSPs were present in both types of regions (Figure 3). Interestingly, when MiSSPs are duplicated, one copy is located in the GDR and one in GSR. Two duplications likely occurred at the same event since the genes were neighbors in both compartments but with invaded repeats in the repeat-rich region (Supplementary Figure S6).

### MiSSP encoding genes show presence–absence polymorphism across *Cenococcum geophilum* isolates

**Figure 4:**
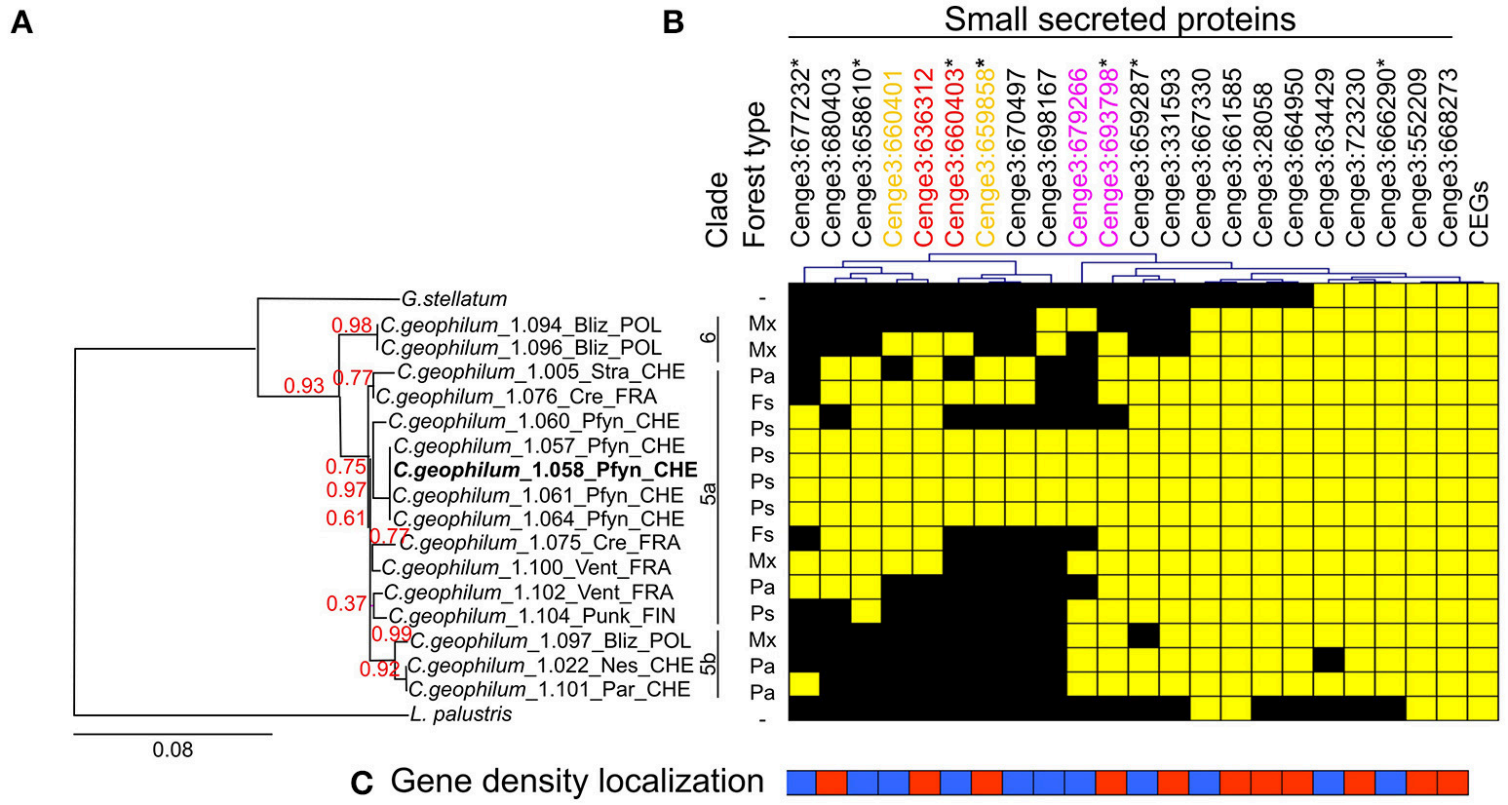
Distribution of genes encoding SSPs among *Cenococcum geophilum* isolates and two closely related species, *Glonium stellatum* and *Lepidopterella palustris*. **(A)** Phylogenetic tree reconstructed based on concatenated nucleotide sequence of the internal transcribed sequence (ITS) and glyceraldehyde-3-phosphate dehydrogenase (GAPDH) gene using PhyML-maximum likelihood. The tree was rooted by *L. palustris* and branch confidence indices were calculated by an approximate likelihood ratio test. The scale bar indicates the number of nucleotide substitutions per site. Three distinct clades are indicated and numbered according to Obase et al. (2016) including a possible subdivision of clade 5 (left). The forest type in which the strains were isolated is indicated as *Picea abies* (Pa), *Pinus sylvestris* (Ps), *Fagus sylvatica* (Fs) and Mixed Forest (Mx). **(B)** Presence (yellow) or absence (black) of genes are indicated for 22 MiSSPs and 22 core eukaryotic genes (CEGs) across the *C. geophilum* strains and the two closely related species. **(C)** Gene density localization is indicated for 22 MiSSPs present in gene sparse (blue) or gene dense region (red). Asterisks next to the Protein IDs indicate that the presence–absence was confirmed by PCR. Presence–absence patterns are hierarchically clustered (top). Color code for gene duplication was indicated (top).

Re-sequencing data from *C. geophilum* isolates originating from different European countries (Switzerland, France, Poland and Finland) and genome data of closely related species, *Glonium stellatum* and *Lepidopterella palustris* allowed to compare SSP sequences for polymorphism (presence–absence) among fungal strains. For seven MiSSPs, PCR amplifications were performed to verify the presence or absence and PCR products were sequenced. Six *C. geophilum* MiSSPs (Cenge3:666290, Cenge3:723230, Cenge3:664950, Cenge3:28058, Cenge3:661585 and Cenge3:667330) were present in all *C. geophilum* isolates but were missing in either *G. stellatum* or *L. palustris* or in both (Figure 4, Supplementary Table S9). Two MiSSPs (Cenge3:668273 and Cenge3:552209) and all analyzed CEGs were present in all *C. geophilum* isolates as well as in *G. stellatum* and *L. palustris*. All other MiSSPs were dispersed among *C. geophilum* isolates. Most conserved MiSSPs (6 of 8; 75%), i.e., those that are present in all *C. geophilum* strains, localized in GDR, whereas only 36% (5 of 14) of the non-conserved MiSSPs did (Figure 4C). The entire set of 22 MiSSPs were present in only three of the four isolates originating from the same site as the sequenced strain. Based on phylogeny analysis using the internal transcribed spacer (ITS) and the glyceraldehyde-3-phosphate dehydrogenase (GAPDH) as marker genes (Obase et al., 2016), these three strains were closely related to the sequenced one and clustered within the clade 5 according to the nomenclature of Obase et al. (2016) (Figure 4A; Supplementary Figure S7). The clades 5 and 6 likely correspond to cryptic species and within clade 5, even more subdivisions are indicated based on species delimitation analyses (Obase et al., 2016; here divided in clade 5a and 5b). When looking at similarities in presence–absence of the 22 MiSSPs among the 16 *C. geophilum* isolates, clade affiliation explained best the polymorphism, whereas country origin marginally and the forest type (and therefore potential plant host) did not significantly explain these patterns (Supplementary Figure S8).

### *C. geophilum* MiSSPs accumulate in distinct plant subcellular compartments

**Figure 5:**
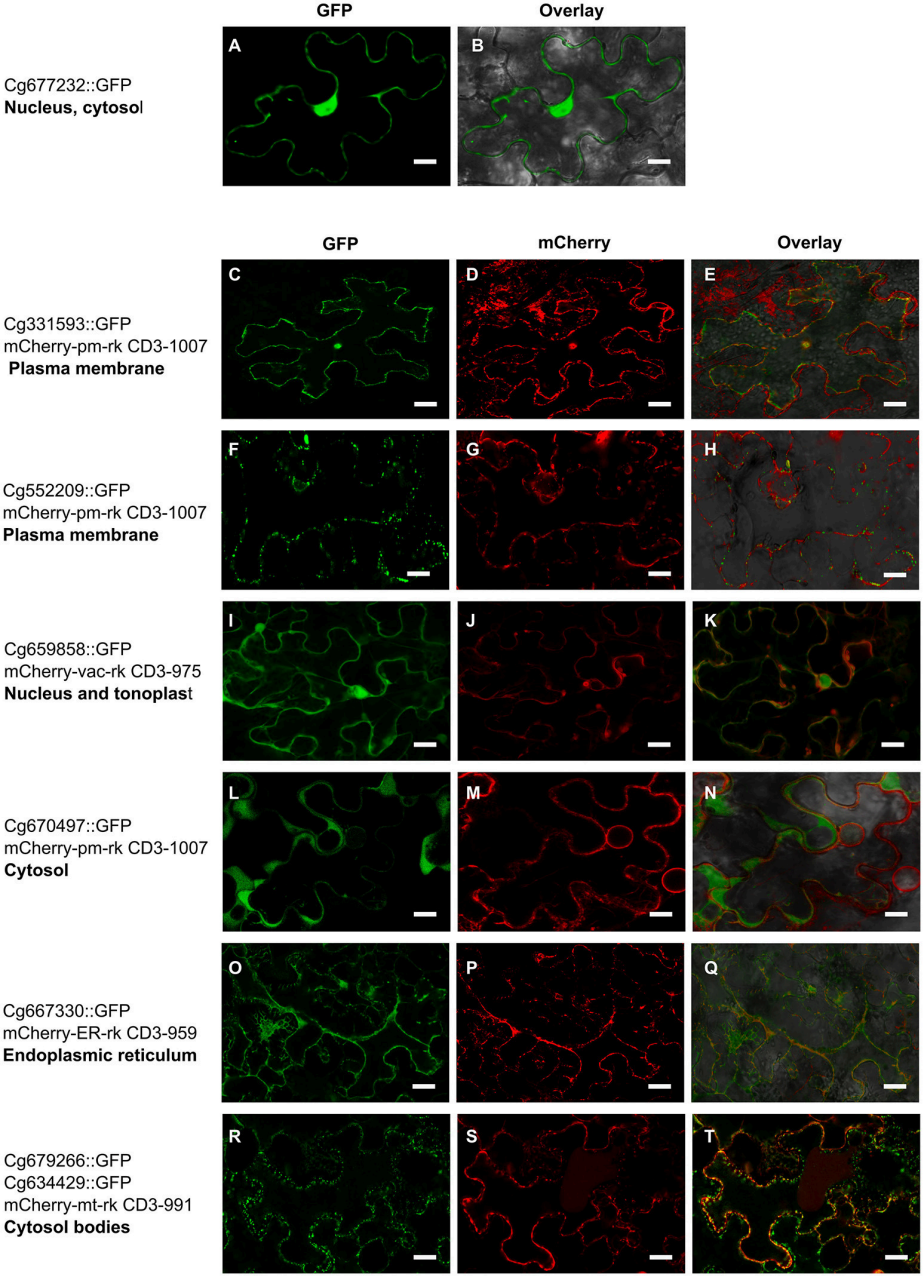
*Cenococcum geophilum* MiSSP candidates accumulate in different subcellular compartments Live cell imaging of six MiSSP:GFP fusion proteins accumulating in specific organelle localization. For each MiSSP:GFP fusion proteins tGFP, mCherry and the overlay are shown. **(A–B)** A representative image for fusion proteins accumulating in the nucleoplasm and cytosol is shown. **(C–H)** Plasma membrane, **(I–K)** tonoplast, **(L–M)** cytosol, **(O–Q)** endoplasmic reticulum and **(R–T)** cytosolic bodies in *Nicotiana benthamiana* leaf cells.

To determine possible *in planta* subcellular location of the 22 *C. geophilum* MiSSP, we cloned their coding DNA sequence (CDS) without the signal peptide (mature form of protein) in an expression vector to obtain MiSSPs fused to a green fluorescent protein (GFP) and expressed them into tobacco leaf cells. 21 *C. geophilum* MiSSP::GFP fusions emitted a detectable fluorescent signal using confocal microscope (Figure 5). The fluorescent signals of Cenge3:552209-GFP, Cenge3:667330-GFP, and Cenge3:659858-GFP accumulated in the plasma membrane, endoplasmic reticulum, and tonoplast, respectively. The signals of Cenge3:679266-GFP and Cenge3:634429-GFP accumulated in small cytosolic bodies (Figure 5). The displayed fluorescent signal in specific subcellular compartments was markedly different from GFP controls and the localization was confirmed by co-expression of specific organelle plant markers (Nelson et al., 2007; Figure 5). All other *C. geophilum* SSP-GFP showed an uninformative subcellular distribution in the nucleus and cytosol as did the GFP control (Supplementary Figure S9). It is important to consider that these localizations were obtained using a 35S promoter and GFP (a protein with triple size of our protein of interest) as a tag. Both actions could result to different localization from those observed when MiSSPs are delivered by the symbiont. Immunoblotting experiments demonstrated both protein production and the integrity for 20 fusion proteins displayed a band at the expected protein size, confirming their integrity. In contrast, one fusion protein (Cenge3:698167) showed no detectable fluorescent signal and no bands on the immunoblots and another (Cenge3:679266) was localized at cytosolic bodies but the integrity could not be confirmed (Supplementary Figure S10). In conclusion, we showed that four distinct subcellular compartments are targeted by the selected *C. geophilum* MiSSPs, including the plasma membrane, tonoplast, cytosol and endoplasmic reticulum.

## Discussion

*Cenococcum geophilum* is a cosmopolitan ECM fungus well known for its extremely wide range of host plants and habitats (LoBuglio, 1999). Although some intraspecific variation in host specificity may exist among different isolates of *C. geophilum* as indicated by a re-synthesis experiment of this species (Antibus et al., 1981) and as known for other generalist ECM species (Le Quéré et al., 2004), the studied *C. geophilum* 1.58 isolate forms ECMs with diverse hosts both gymnosperms (*Pinus sylvestris, Picea abies*) and angiosperms (*Populus* spp., *Quercus* spp.; M. Peter unpublished). To understand its communication strategy with different symbiotic partners, we compared the gene regulation of secreted proteins and found that the same gene sets were used and similarly expressed in ECMs of *C. geophilum* formed with either host. Only very few genes were differentially expressed, which is astonishing for so different trees as are the gymnosperm pine and the angiosperm poplar. Only few studies about host specific interactions are available from ECM systems. The generalist *Laccaria bicolor* also expressed a core gene regulon when interacting with the two different hosts *Pseudotsuga menziesii* and *Populus trichocarpa* but almost 80% of the about 4,000 up-regulated genes were specific to one of the host trees, among which many secreted proteins (Plett et al., 2015). On the contrary, a very similar set of genes was expressed in compatible interactions between species of the *Suillus* genus, considered as specialists on *Pinaceae*, interacting with different *Pinus* species and significant differences were mainly observed in incompatible interactions (Liao et al., 2016). Generalists such as the root endophyte *Pirifomospora indica* (Lahrmann et al., 2013) and the plant pathogen *Sclerotinia sclerotiorum* (Guyon et al., 2014) clearly induced host-specific gene sets whereas the specialist barley powdery mildew pathogen *Blumeria graminis* f. sp. *hordei* expressed very similar gene sets when interacting with divergent hosts such as monocots and dicots (Hacquard et al., 2013). Being a very broad generalist, we therefore expected *C. geophilum* to rather show a host-specific regulon, which was not the case with only 8% of all up-regulated genes being host-specific. More work is needed to see whether such a uniform response of *C. geophilum* holds true for other host trees and in more natural systems such as in greenhouse trials. The small set of differentially regulated genes likely corresponds to the fine-tuning necessary for the interaction with each host tree. A more than five-fold higher difference in transcript abundance was detected mainly for CAZymes during the interaction between *C. geophilum* and pine and for MiSSPs in *C. geophilum* poplar ECM. Since pine and poplar roots have different cell wall compositions (Sarkar et al., 2009), a different set of cell-wall loosening enzymes could be necessary for the penetration of hyphae and development of the Hartig net. For instance, the two GH28 acting on pectins, a GH5-4 acting on hemi-/cellulose and a GH45 a cellulase with similarity to plant expansins that have an important role in plant cell wall loosening (Cosgrove, 2000) are among the more highly expressed genes in pine ECM. The colonization of the gymnosperm *P. menziesii* by *L. bicolor* was also accompanied by a high number of differentially expressed CAZymes with increased abundances; among these were GH5 and GH28. On the opposite, *L. bicolor* interacting with poplar roots under semi-sterile greenhouse conditions expressed a dozen of SSPs specifically in interaction with poplar (Plett et al., 2015), which fits well with our observations.

Expression profiling data from *in vitro* ECM produced in this work revealed that several MiSSPs up-regulated in greenhouse (Peter et al., 2016) were not regulated in the *in vitro* syntheses even when interacting with the same host tree. This suggests that environmental factors are equally important for the regulation of MiSSPs. Proteomic analysis of the secretome of *Hebeloma cylindrosporum* free-living mycelium revealed that 17% of the secreted proteins were SSPs (Doré et al., 2015). These SSP-encoding genes were differentially regulated in ECM root tips and depending on the environmental conditions. Likewise, gene profiling of extramatrical mycelium and sclerotia of *C. geophilum* performed in the present study showed that some MiSSPs were not only up-regulated in the ECM root tip itself, but also in other fungal tissues in the presence of a host plant, which indicates that they are induced by the host plant but play a role in biological processes not directly linked to the fungal-plant communication at the symbiotic interface. All these data strengthen the concept that fungal SSPs not only play an important role as candidate effector genes in fungal-plant interactions, but also in the adaptation to their environment and saprotrophic growth (Doré et al., 2015).

*C. geophilum* is the first ectomycorrhizal fungus showing a bipartite genome architecture with repeat-rich, gene-poor regions and vice versa. This genome compartmentalization refers to regions with uneven mutation rates, GC-content and gene density with the gene-sparse, repeat-rich compartments evolving at higher rates (Raffaele and Kamoun, 2012; Plissonneau et al., 2017). This phenomenon has first been described for the oomycete *Phytophtora infestans* genome (Haas et al., 2009) and recently, a convergence toward similar genome architecture has been demonstrated in phylogenetically unrelated fungal plant pathogens such as *Leptosphaeria maculans* (Rouxel et al., 2011; Grandaubert et al., 2014) and *Zymoseptoria triticii* (Stukenbrock et al., 2010). In all these plant-pathogens, the two-speed-genome explained genomic plasticity in order to increase and adapt the repertoire of effector/avirulence genes (Möller and Stukenbrock, 2017). The rapidly evolving compartment in pathogen genomes is largely controlled by transposable elements (Plissonneau et al., 2017) being hot spots for duplication, deletion, and recombination as well as local mutagenesis through TE-silencing mechanisms such as repeat induced point mutation (RIP; Dong et al., 2015). The high content of transposable elements in *C. geophilum* (75% of genome; Peter et al., 2016) might therefore also play an important role in genome adaptation and since TEs are arranged in different compartments, these might also evolve at different rates. Moreover, in plant pathogens, genes implicated in virulence and host adaptation such as effector genes tend to localize in repeat-rich, faster evolving regions (Raffaele and Kamoun, 2012). The genes coding for the selected 22 MiSSPs of *C. geophilum* are localized not only in repeat rich but also in gene dense regions. This is true also for other *in planta*-induced genes and differs from what has been found for the pathogenic oomycete *P. infestans* (Raffaele et al., 2010a). We noticed that conserved MiSSPs are rather located in gene-dense regions, whereas those that are dispersed among *C. geophilum* isolates are more often found close to repeats. This indicates that these MiSSPs might evolve at different rates. Likewise, duplicated MiSSPs that each have a member in both regions show different presence–absence patterns among *C. geophilum* isolates, and are therefore evolving differentially. Subcellular localization analyses for one of the three duplications indicate that the duplicated genes might have different functions since one member targeted the tonoplast within the plant cell (Cenge3:659858) whereas the other showed no specific localization (Cenge3:660401). Interestingly, gene expression and regulation for duplicated genes located in either repeat-rich regions or gene-dense regions were different, indicating that TEs might affect promotor regions and thereby gene regulation and/or that duplicated genes play different roles in the fungal-host interaction. Clearly more population genomic and functional studies are needed to elucidate the evolutionary rate of change of these duplications and its functional significance for adaptation.

Intra- and interspecies comparisons of MiSSP presence revealed that some MiSSPs are conserved not only in *C. geophilum* isolates but also in saprotrophic relatives. Distinct factors can contribute to secretome variation and evolution such as host specificity, phylogenetic history and lifestyle (Kohler et al., 2015; Pellegrin et al., 2015; Liao et al., 2016; Kamel et al., 2017). The genomes of ECM fungi analyzed so far, share a large set of SSPs with brown and white rot fungi and litter decayers (Kohler et al., 2015; Pellegrin et al., 2015; Martin et al., 2016) and these are likely involved in conserved processes such as developmental changes (e.g., hyphal aggregation in mycelia and fruiting body formation), saprotrophic growth or soil environmental interactions, as indicated by the present and other gene expression studies (Doré et al., 2015). Further, intraspecific analyses show that several species-specific MiSSPs found in the reference genome 1.58 are absent in other *C. geophilum* strains. This dispersion of MiSSPs within *C. geophilum* isolates, which to some extend reflect phylogenetic sub-clades, suggests that gene gain and loss may be an important driver of evolution as shown for pathogenic fungi (Syme et al., 2013; van Dam et al., 2016; Hartmann and Croll, 2017). Whether sub-clades identified with commonly used phylogenetic marker genes do reflect cryptic species, needs to be evaluated using genome-wide polymorphism analyses of additional isolates of *C. geophilum* populations. Likewise, the role mating, but also TE activity, which has been suggested as possible driver of cryptic speciation in plant systems (Bonchev and Parisod, 2013), play in microevolutionary processes within this taxon remains to be determined.

Over the past 5 years, information about secretome repertoires with a particular emphasis on SSPs became available for fungi with different ways of life (Guyon et al., 2014; Lo Presti et al., 2015; Pellegrin et al., 2015; Kamel et al., 2017). However, only a few studies provide data regarding the role of these fungal SSPs in mutualistic plant-microbe interactions. One limitation is that the majority of SSPs are orphan genes that have no domains of known function. Tools to assess their functional role are scarce for species that cannot be transformed and easily handled in the laboratory, as are mycorrhizal fungi. One available approach to elucidate their role is to first identify their subcellular localization in an heterologous system and then try to identify their potential targets (Alfano, 2009). Confocal microscopy assays revealed that five *C. geophilum* MiSSPs over the 22 tested target distinct sub-cellular compartments, such as plasma membrane, cytosol, endoplasmic reticulum and tonoplast.

Only six MiSSPs showed known domains or homology to other proteins. For example, the cupin domain containing MiSSP, which was conserved in all fungal strains studied here, localized to the plasma membrane and was only induced in ECM of pine. This domain is found in a wide variety of functionally diverse proteins in eukaryotes and prokaryotes (Dunwell et al., 2000) which does not allow speculating about possible functions of this MiSSP. A second MiSSP contains a NTF2 super family domain, probably similar to the one in SnoaL polyketide cyclase. These domains are found in several organisms, including filamentous fungal phytopathogens and present different functions within the proteins, including both enzymatic and non-enzymatic versions (Eberhardt et al., 2013; Deng et al., 2017). This MiSSP was conserved among *C. geophilum* isolates and *L. palustris* and localized in plant endoplasmic reticulum. The third MiSSP was the most highly expressed and up-regulated (Cenge3:698167) in ECM roots and to a lower degree in root-associated sclerotia in all ECM experiments. Unfortunately, sub-cellular localization experiments were unsuccessful for this particular MiSSP. It shows similarity (66%) to the “secreted in xylem 1” (Six1) effector of the asexual, soil inhabiting ascomycete *Fusarium oxysporum* that can switch from a saprotrophic to a pathogenic lifestyle infecting plant roots (Rep et al., 2004). Although several studies have been performed on this protein, the exact function of it is still unclear. This protein (and homologs of it) has been shown to be secreted in root xylem vessels, the gene expression being induced in early root infection only by living cells and that it plays a role in virulence (Rep et al., 2004; Van Der Does et al., 2008; Li et al., 2016). Only strains of the polyphyletic formae speciales lycopersici causing tomato wilt have the genomic region containing Six1 (Van Der Does et al., 2008). In *C. geophilum*, this MiSSP was only present in a few strains and it remains to be determined, whether it is highly expressed in ECM roots of all these strains and what role it could play in the symbiotic fungal-plant interaction.

None of the MiSSPs analyzed in this work were predicted to localize in the nucleus. We expected such a localization as the two symbiotic effectors characterized in mycorrhizal fungi so far, MiSSP7 and SP7, and many effectors from pathogens target the nucleus (Kloppholz et al., 2011; Plett et al., 2011; Petre et al., 2015). Two MiSSPs (Cenge3:679266 and Cenge3:634429) formed cytosolic bodies and the irregularity of the bodies suggesting that they might be artefactual aggregates, as proposed for localization of rust effectors (Petre et al., 2015; Qi et al., 2018). It is important to consider that these localizations were obtained using GFP as a tag, which can interfere with MiSSPs localization as it is a large fluorescent tag of ~27 kDa (Varden et al., 2017). However, the proportion of informative localizations as well as identified compartments are consistent with similar studies on SSPs of filamentous plant-pathogenic fungi (Caillaud et al., 2012; Chaudhari et al., 2014; Petre et al., 2015, 2016; Germain et al., 2017; Varden et al., 2017; Qi et al., 2018). Next steps now are to localize the MiSSPs in root cells of host plants and to search for direct plant targets using co-immunoprecipitation/mass spectrometry. The confirmation that a MiSSP is an authentic symbiotic effector requires the demonstration that it is essential for symbiosis development and that it has the ability to interfere with a host component to conclusively support symbiosis. A promising approach in this respect is to use double stranded interfering (dsi) RNA to knock down transcription of MiSSPs, a method successfully applied in fungal-plant systems for which efficient transformation protocols are lacking (Wang et al., 2016).

## Author contributions

Conceived and designed the experiments: MP, AK, CV-F, FM, MdFP, and KB; Performed the experiments: MdFP, PV, FG, SP, HN, and MK; Analyzed sequence data: MdFP, EM, AK, CV-F, VS, AL, and MP; Drafted the manuscript: MdFP, AK, CV-F, and MP; Revised the manuscript: MdFP, MP; AK, CV-F, SE, IG, and FM. All authors read and approved the final manuscript.

### Conflict of interest statement

The authors declare that the research was conducted in the absence of any commercial or financial relationships that could be construed as a potential conflict of interest.

## Supplementary material

Supplementary Figure S1 Ectomycorrhiza formed by *Cenococcum geophilum* and their host plants. Morphological characteristics of typical ectomycorrhiza formed by *C. geop*hilum in interaction with *Pinus sylvestris* **(A)** and *Populus tremula L*. × *Populus alba* L.-INRA 717) **(C)**. Cross-sections of ectomycorrhiza roots of both system shows a presence of the Hartig net between epidermal and cortex cells in both interactions **(B,D)**. Percentage of ectomycorrhiza formation between *C. geophilum* and *P. sylvestris* and *C. geophilum* and *P. tremula* × *P. alba* **(E)**.

Supplementary Figure S2 Comparison of *C. geophilum* gene expression changes in greenhouse pine ECM, *in vitro* pine ECM and *in vitro* poplar ECM. Venn diagram based on the comparison of secreted proteins regulated in at least one experiment: ECM formed by *C. geophilum* with *Pinus sylvestris* [*in vitro* synthesis or greenhouse (Peter et al., 2016)] and *C. geophilum* with *Populus tremula* × *Populus alba* L.-INRA 717 (*in vitro* synthesis). Note that the age of control mycelium was different: *C. geophilum* with *P. sylvestris* [*in vitro* synthesis = 90 days, *C. geophilum* with *P. sylvestris* greenhouse = 15 days; *C. geophilum* with *Populus tremula* × *P. alba in vitro* synthesis = 60 days. Data are provided in Supplementary Table S6.

Supplementary Figure S3 Nucleotide **(A–C)** and protein **(D–F)** alignments of duplications of candidate MiSSPs in the *C. geophilum* genome. **(A,D)** Cenge3:636312 and Cenge3:660403, **(B,E)** Cenge3:679266 and Cenge3:693798, **(C,F)** Cenge3:660401 and Cenge3:659858. Protein ID from Joint Genome Institute (JGI).

Supplementary Figure S4 Distribution of gene expression induction in ectomycorrhizal root tips compared to free-living mycelium according to local gene density for all genes. The median **(A)**, minimum **(B)** or maximum **(C)** induction (log_2_ ratio ECM vs. FLM) values associated to genes in each bin are shown as a color-coded heat map. **(D)** Distribution of the average gene expression level in ectomycorrhizal root tips according to local gene density. The median values for gene expression in each bin are shown as a color-coded heat map. Data are presented for ECM root tips of *C. geophilum* and *P. sylvestris*- semi-sterile under greenhouse conditions (left column) or *in vitro* system (middle column) and for *C. geophilum- Populus tremula x alba in vitro* (right column).

Supplementary Figure S5 Percentage and number of genes found in gene-dense repeat sparse or gene sparse repeat rich regions for the proteome and the secretome of *C. geophilum*. The secretome was categorized into functional categories (proteases, lipases, CAZymes, SSPs and other secreted proteins). Enrichment tests were not significant.

Supplementary Figure S6 Genomic landscape of compartments on scaffold 21 and 23 of *Cenococcum geophilum* harboring duplications of MiSSPs in gene-dense and gene-poor, repeat-rich regions. Displays are extracted from the genome viewer of the Joint Genome Institute (JGI) website (https://genome.jgi.doe.gov/Cenge3/Cenge3.home.html) showing tracks of base position, GC content, predicted genes (GeneCatalog; dark blue), and predicted repetitive regions (black, 3 tracks) discovered by RepeatScout and masked by RepeatMasker.

Supplementary Figure S7 Phylogenetic tree of *C. geophilum* strains and the closest relative *Glonium stellatum* reconstructed based on concatenated nucleotide sequences of the internal transcribed spacer (ITS) and the glyceraldehyde-3-phosphate dehydrogenase (GAPDH) using PhyML-maximum likelihood. In addition to the 15 *C. geophilum* strains from the present study, six representative strains of the six clades from the study of Obase et al. (2016) were included in the analysis. Branch confidence indices were calculated using an approximate likelihood ratio test. The scale bar indicates the number of nucleotide substitutions per site. Three distinct clades are indicated and numbered according to Obase et al. (2016) including a possible subdivision of clade 5 (left). *Glonium stellatum* was designated as the outgroup.

Supplementary Figure S8 Variability in presence/absence of 22 MiSSP genes among 16 *C. geophilum* isolates. The first two axes of a principal coordinate analysis based on the Jaccard similarity index are provided. Each symbol represents an isolate originating from the given country with isolates closer to each other showing more similar presence/absence patterns. In **(A)**, different symbols indicate the phylogenetic clade the isolate are grouped into based on a concatenated dataset of the ITS and GADPH regions (Obase et al., 2016). In **(B)**, different symbols indicate the forest type with the dominating tree species: Mx, mixed forest; Pa, *Picea abies*; Fs, *Fagus sylvatica;* Ps, *Pinus sylvestris*. **(C)** PERMANOVA table showing the effects of phylogenetic clade, country of origin and forest type of isolation on the MiSSP presence/absence patterns in the 16 *C. geophilum* isolates. Analyses were performed with the Primer-E software (Clarke and Gorley, 2015).

Supplementary Figure S9 Candidate effectors with no informative localization *in planta*. Representative images corresponding to the 13 fusion proteins accumulating in the nucleoplasm and the cytosol. The fusion proteins were transiently expressed in *Nicotiana benthamiana* leaf cells by agroinfiltration. Live-cell imaging was performed with a laser-scanning confocal microscope 2 days after infiltration. The green fluorescent protein (GFP) was excited at 488 nm. GFP (green) fluorescence was collected at 505–525 nm.

Supplementary Figure S10 Immunoblots of CgMiSSPs:GFP fusion proteins in *N. benthamiana* leaves. GFP detection was performed in a single step by a GFP-HRP conjugated antibody. The theoretical size of each fusion protein (SSP+GFP) is indicated between parentheses in kiloDalton (kDa). Page rulers and corresponding sizes in kiloDalton (kDa) are indicated on the blots. White asterisks indicate specific protein bands.

Supplementary Table S1 *Cenococcum geophilum* and other fungal strains used in this work.

Supplementary Table S2 Main features of *C. geophilum* RNAseq data.

Supplementary Table S3 Main features of *C. geophilum* re-sequencing data.

Supplementary Table S4 Core eukaryotic genes selected for presence/absence polymorphism analysis.

Supplementary Table S5 Concatenated and separated sequences of *C. geophilum* glyceraldehyde 3-phosphate dehydrogenase (GAPDH) and internal transcribed spacer (ITS).

Supplementary Table S6 List of primers used to amplify seven MiSSPs in *C. geophilum* mycelium.

Supplementary Table S7 Genes encoding small secreted proteins regulated in the interaction between *Cenoccoccum geophilum* and *Pinus sylvestris* or *Populus tremula* × *alba* as compared to free-living mycelium (FLM).

Supplementary Table S8 Expression comparison of genes encoding secreted proteins significantly regulated in the interaction between *Cenococcum geophilum* and *Pinus sylvestris* or *Populus tremula* × *alba*. A fold change between Pine and Poplar ECM was calculated and a cut-off 5 fold was established to show specificity expression in each tissue.

Supplementary Table S9 Blast results of *C. geophilum* 1.58 MiSSP candidates against de novo assemblies of *C. geophilum* strains. The genes that are absent in respective strains based on e-value and HSP length are marked in red. A fasta file with respective contigs is available (Supplementary File 1).

Supplementary File 1 *C. geophilum* nucleotide sequences contig containing MiSSPs of respective *C. geophilum* strains.

## Abbreviations

CAZymes: Carbohydrate-Active Enzymes
CBM: Carbohydrate binding modules
CDS: Coding DNA sequence
CEG: Core eukaryotic genes
ECM: Ectomycorrhiza
ER: Endoplasmic reticulum
FDR: False discovery rate
FIR: Intergenic flanking region
FPKM: Fragment Per Kilobase of exon model per Million mapped reads
GAPDH: Glyceraldehyde-3-phosphate dehydrogenase
GEO: Gene Expression Omnibus
GFP: Green fluorescent protein
GH: Glycoside hydrolase
GDR: gene-dense repeat-poor region
GSR: gene-sparse repeat-rich region
GT: glycosyl transferase
ITS: Internal transcribed spacer
MiSSP: Mycorrhizae induced Small Secreted Protein
SP: Secreted Protein
SiP: Signal Peptide
SSP: Small Secreted Proteins
TE: Transposable Elements
TPM: Transcripts per million.
