# Supplementary figures and images for: Secretome Analysis from the Ectomycorrhizal Ascomycete Cenococcum geophilum
Source: Front Microbiol. 2018 Feb 13;9:141. doi: 10.3389/fmicb.2018.00141 (PMC5816826; doi:10.3389/fmicb.2018.00141)

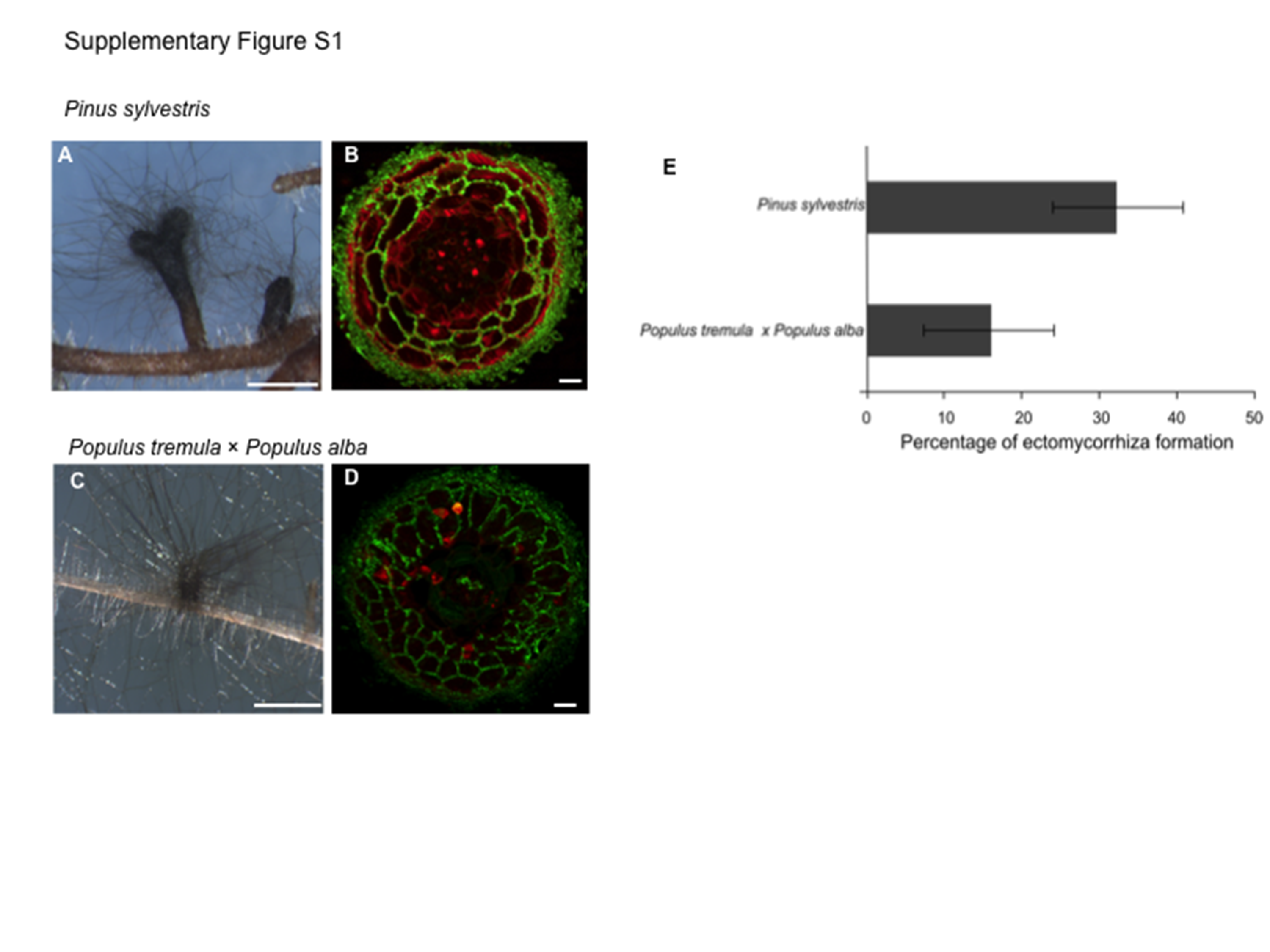

Supplement: Supplementary Figure S1 — Ectomycorrhiza formed by Cenococcum geophilum and their host plants. Morphological characteristics of typical ectomycorrhiza formed by C. geophilum in interaction with Pinus sylvestris (A) and Populus tremula L. × Populus alba L.-INRA 717) (C). Cross-sections of ectomycorrhiza roots of both system shows a presence of the Hartig net between epidermal and cortex cells in both interactions (B,D). Percentage of ectomycorrhiza formation between C. geophilum and P. sylvestris and C. geophilum and P. tremula × P. alba (E). [file Image1.TIFF]

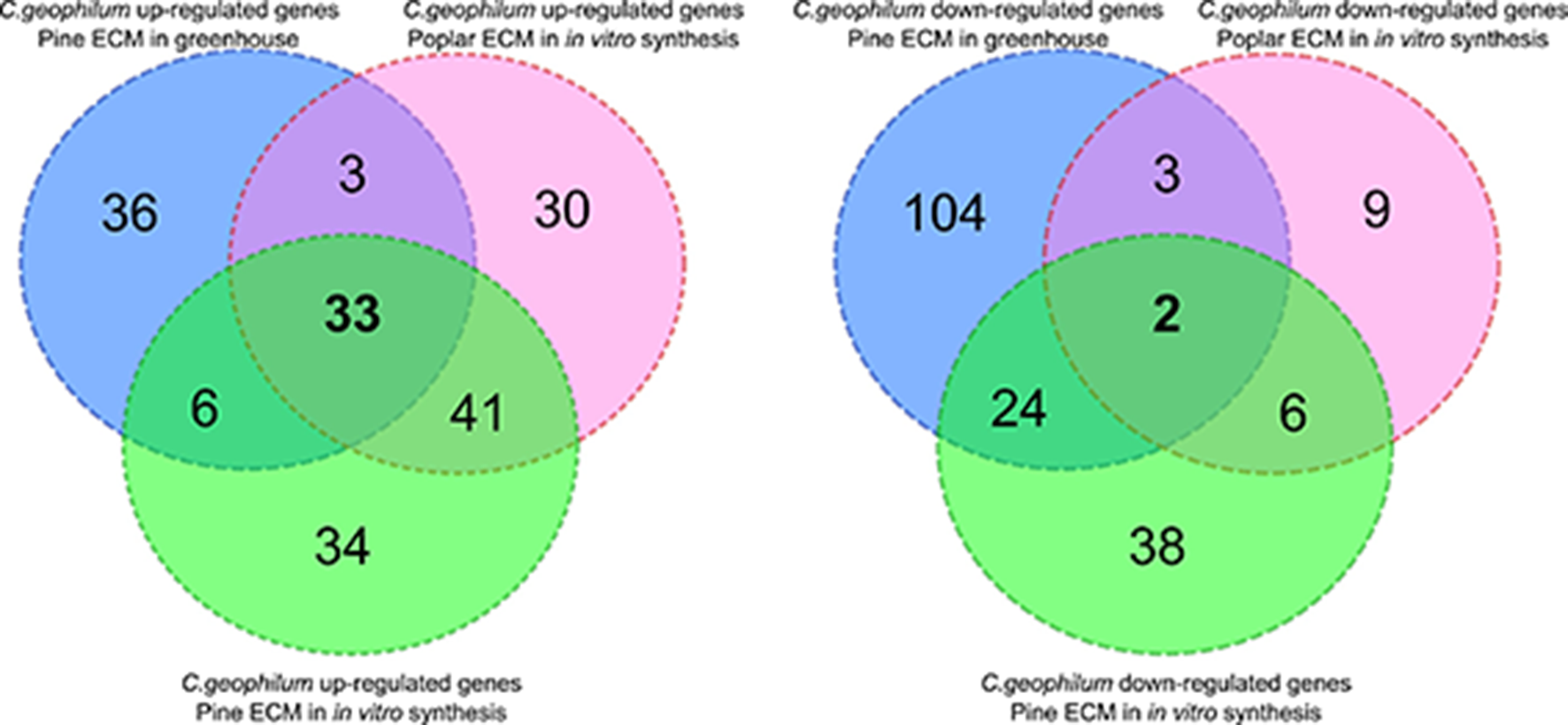

Supplement: Supplementary Figure S2 — Comparison of C. geophilum gene expression changes in greenhouse pine ECM, in vitro pine ECM and in vitro poplar ECM. Venn diagram based on the comparison of secreted proteins regulated in at least one experiment: ECM formed by C. geophilum with Pinus sylvestris [in vitro synthesis or greenhouse (Peter et al., 2016)] and C. geophilum with Populus tremula × Populus alba L.-INRA 717 (in vitro synthesis). Note that the age of control mycelium was different: C. geophilum with P. sylvestris [in vitro synthesis = 90 days, C. geophilum with P. sylvestris greenhouse = 15 days; C. geophilum with Populus tremula × P. alba in vitro synthesis = 60 days. Data are provided in Supplementary Table S6. [file Image2.TIFF]

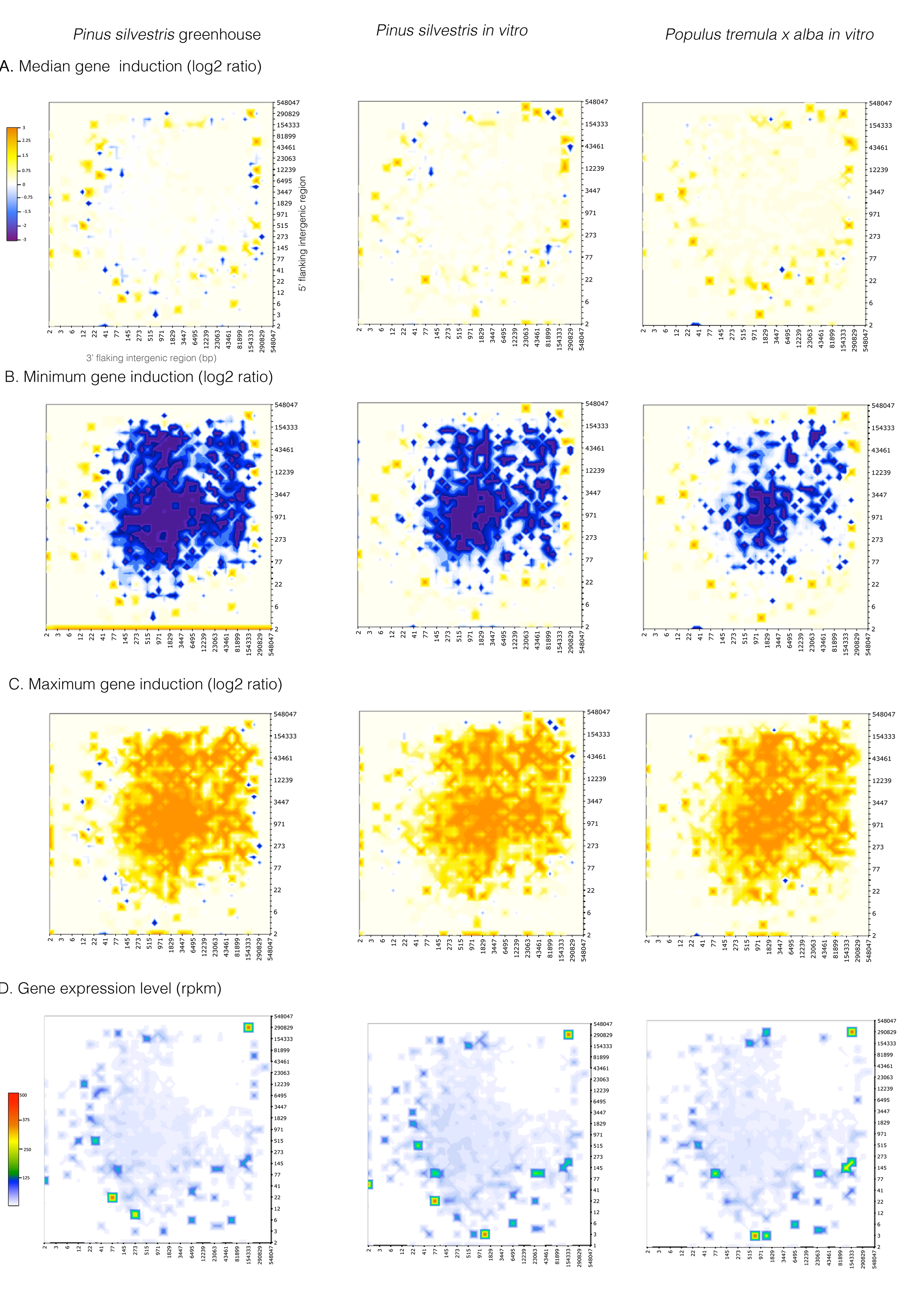

Supplement: Supplementary Figure S4 — Distribution of gene expression induction in ectomycorrhizal root tips compared to free-living mycelium according to local gene density for all genes. The median (A), minimum (B) or maximum (C) induction (log2 ratio ECM vs. FLM) values associated to genes in each bin are shown as a color-coded heat map. (D) Distribution of the average gene expression level in ectomycorrhizal root tips according to local gene density. The median values for gene expression in each bin are shown as a color-coded heat map. Data are presented for ECM root tips of C. geophilum and P. sylvestris- semi-sterile under greenhouse conditions (left column) or in vitro system (middle column) and for C. geophilum- Populus tremula x alba in vitro (right column). [file Image4.TIF]

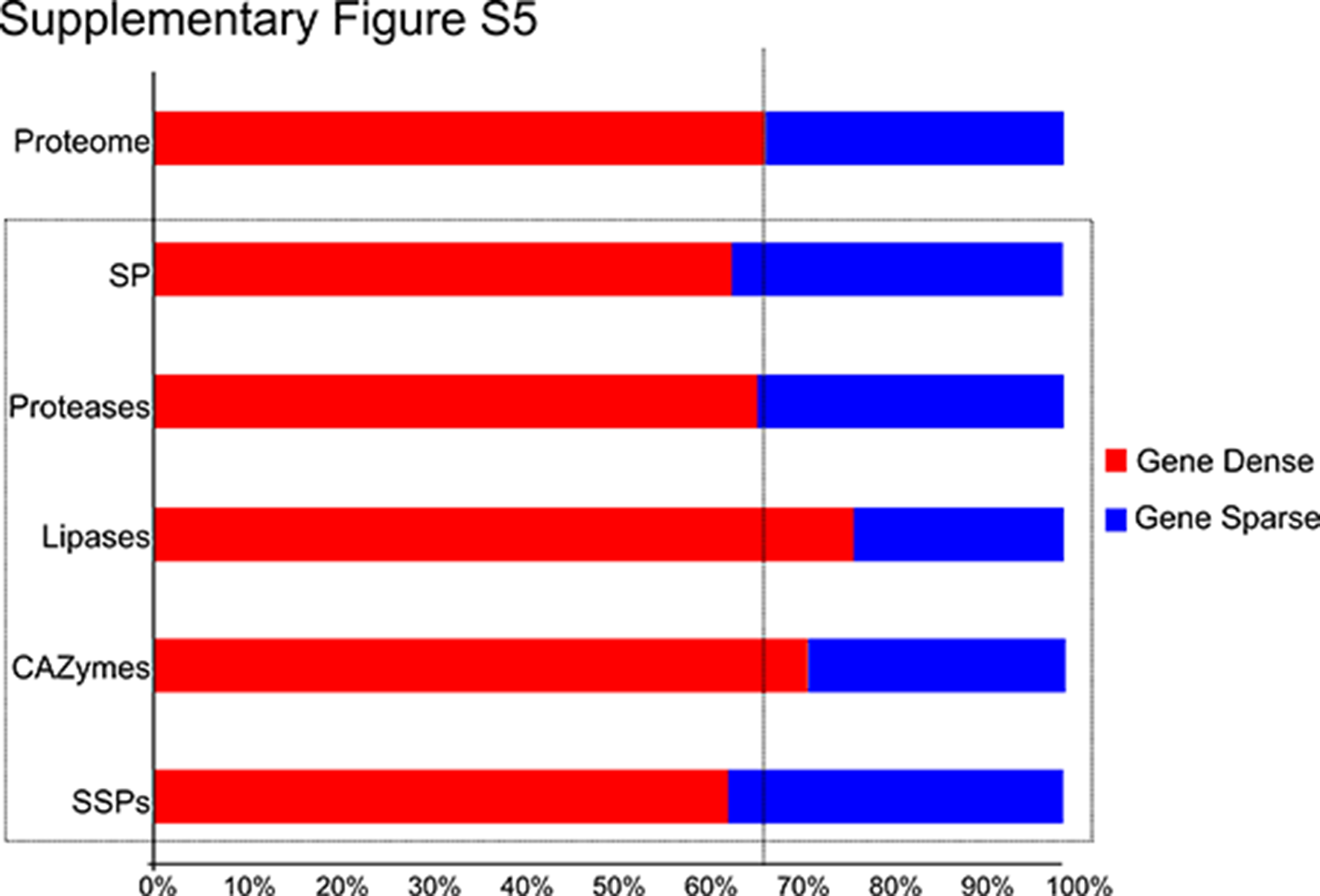

Supplement: Supplementary Figure S5 — Percentage and number of genes found in gene-dense repeat sparse or gene sparse repeat rich regions for the proteome and the secretome of C. geophilum. The secretome was categorized into functional categories (proteases, lipases, CAZymes, SSPs and other secreted proteins). Enrichment tests were not significant. [file Image5.TIFF]

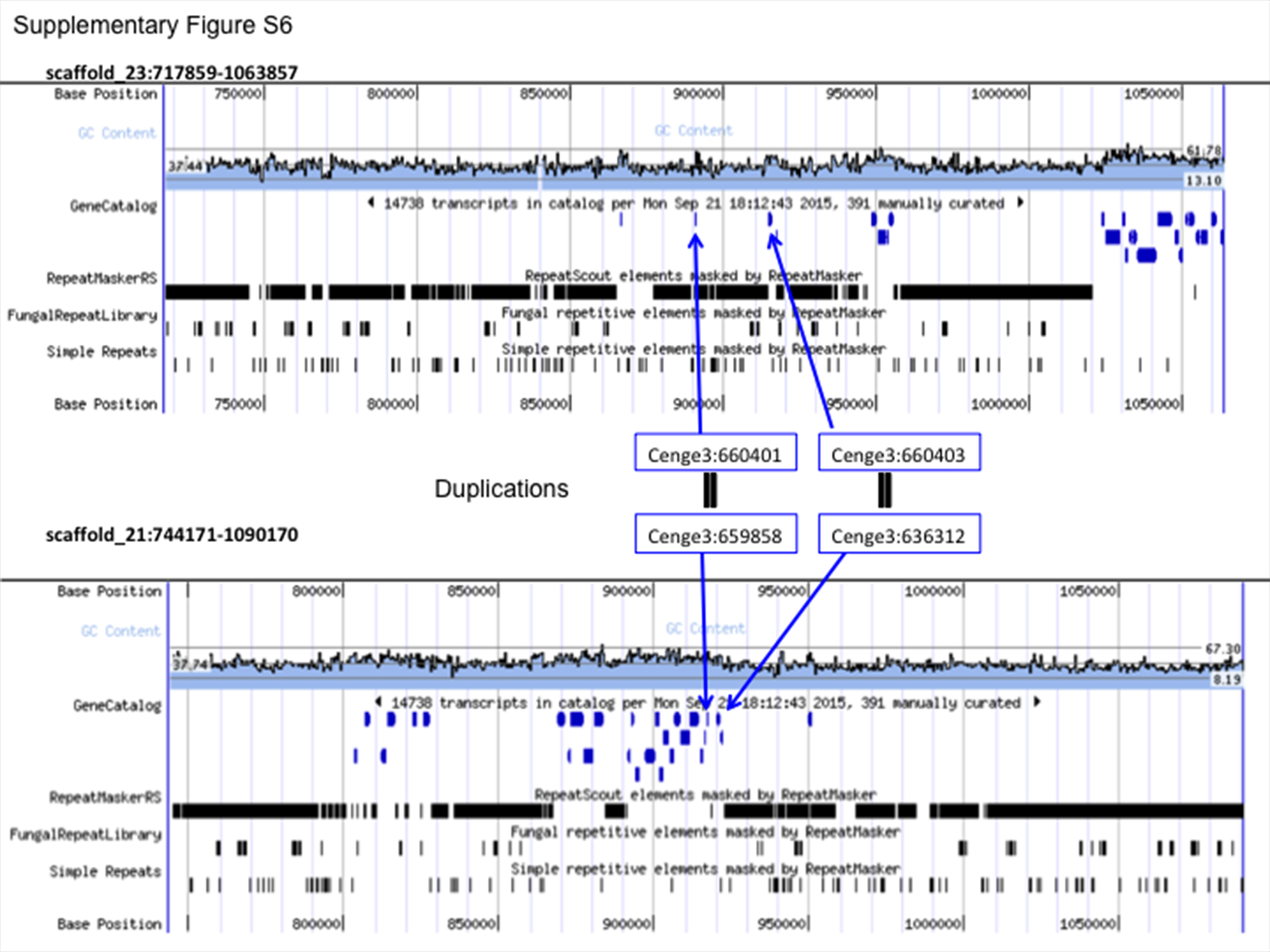

Supplement: Supplementary Figure S6 — Genomic landscape of compartments on scaffold 21 and 23 of Cenococcum geophilum harboring duplications of MiSSPs in gene-dense and gene-poor, repeat-rich regions. Displays are extracted from the genome viewer of the Joint Genome Institute (JGI) website (https://genome.jgi.doe.gov/Cenge3/Cenge3.home.html) showing tracks of base position, GC content, predicted genes (GeneCatalog; dark blue), and predicted repetitive regions (black, 3 tracks) discovered by RepeatScout and masked by RepeatMasker. [file Image6.TIFF]

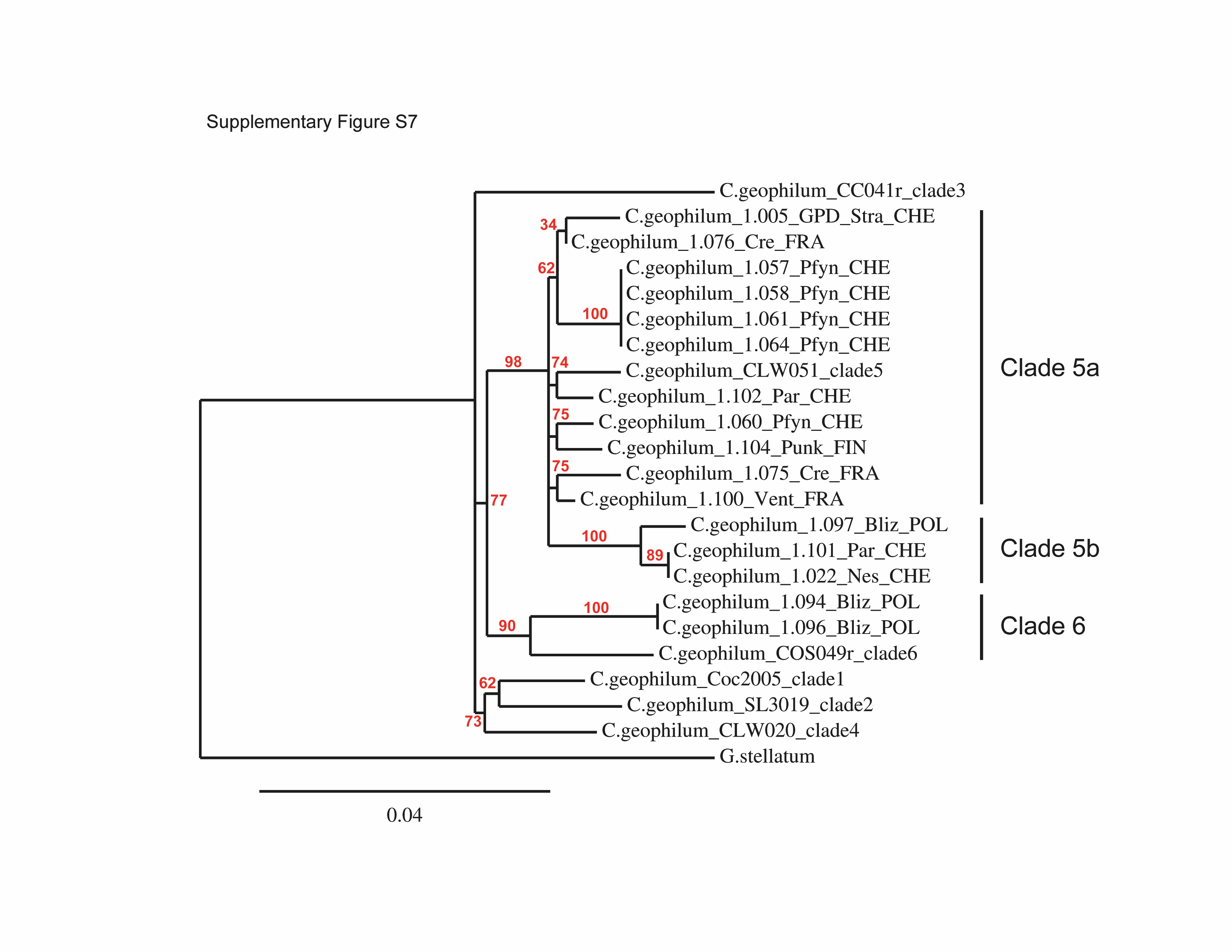

Supplement: Supplementary Figure S7 — Phylogenetic tree of C. geophilum strains and the closest relative Glonium stellatum reconstructed based on concatenated nucleotide sequences of the internal transcribed spacer (ITS) and the glyceraldehyde-3-phosphate dehydrogenase (GAPDH) using PhyML-maximum likelihood. In addition to the 15 C. geophilum strains from the present study, six representative strains of the six clades from the study of Obase et al. (2016) were included in the analysis. Branch confidence indices were calculated using an approximate likelihood ratio test. The scale bar indicates the number of nucleotide substitutions per site. Three distinct clades are indicated and numbered according to Obase et al. (2016) including a possible subdivision of clade 5 (left). Glonium stellatum was designated as the outgroup. [file Image1.png]

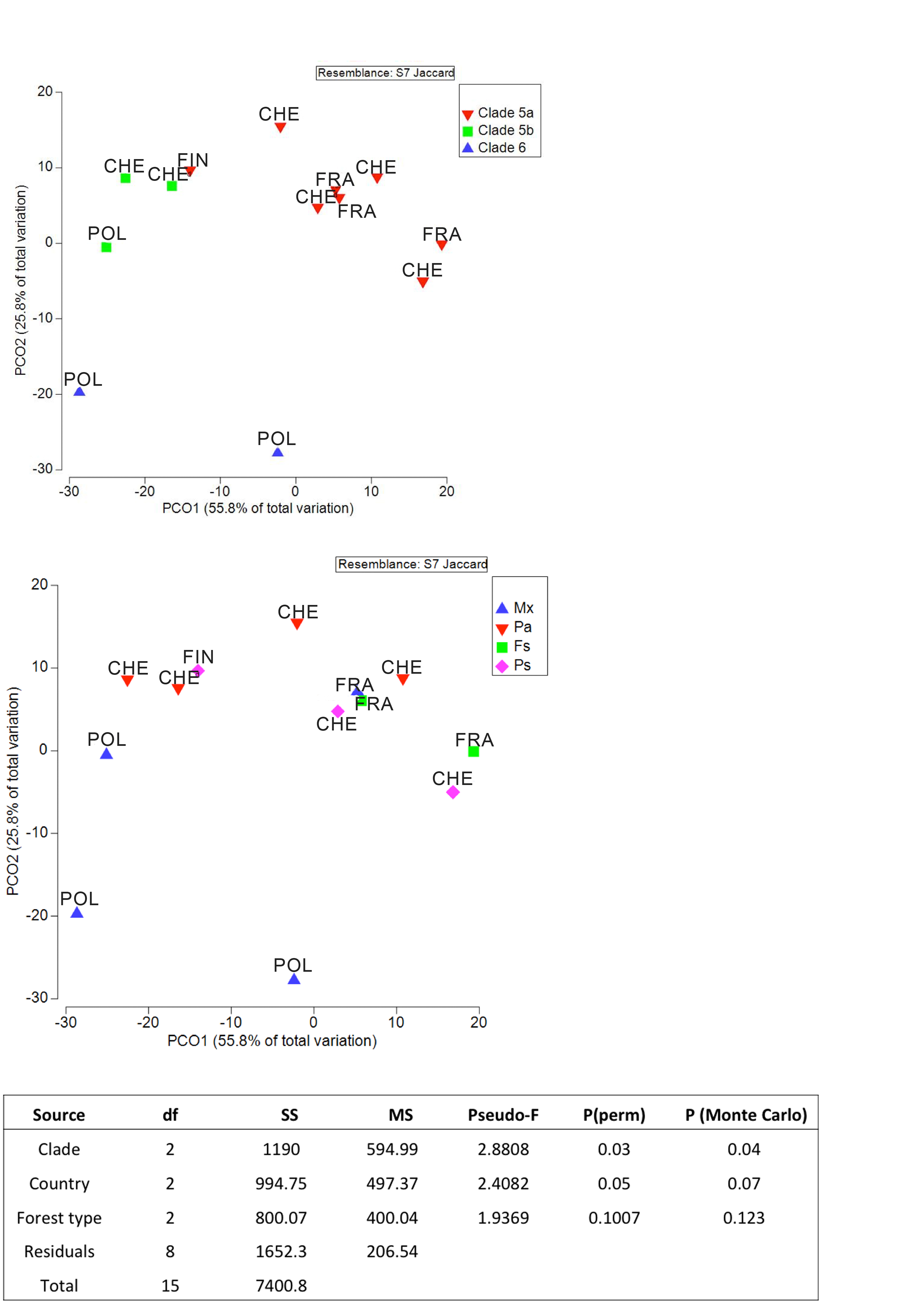

Supplement: Supplementary Figure S8 — Variability in presence/absence of 22 MiSSP genes among 16 C. geophilum isolates. The first two axes of a principal coordinate analysis based on the Jaccard similarity index are provided. Each symbol represents an isolate originating from the given country with isolates closer to each other showing more similar presence/absence patterns. In (A), different symbols indicate the phylogenetic clade the isolate are grouped into based on a concatenated dataset of the ITS and GADPH regions (Obase et al., 2016). In (B), different symbols indicate the forest type with the dominating tree species: Mx, mixed forest; Pa, Picea abies; Fs, Fagus sylvatica; Ps, Pinus sylvestris. (C) PERMANOVA table showing the effects of phylogenetic clade, country of origin and forest type of isolation on the MiSSP presence/absence patterns in the 16 C. geophilum isolates. Analyses were performed with the Primer-E software (Clarke and Gorley, 2015). [file Image8.JPEG]

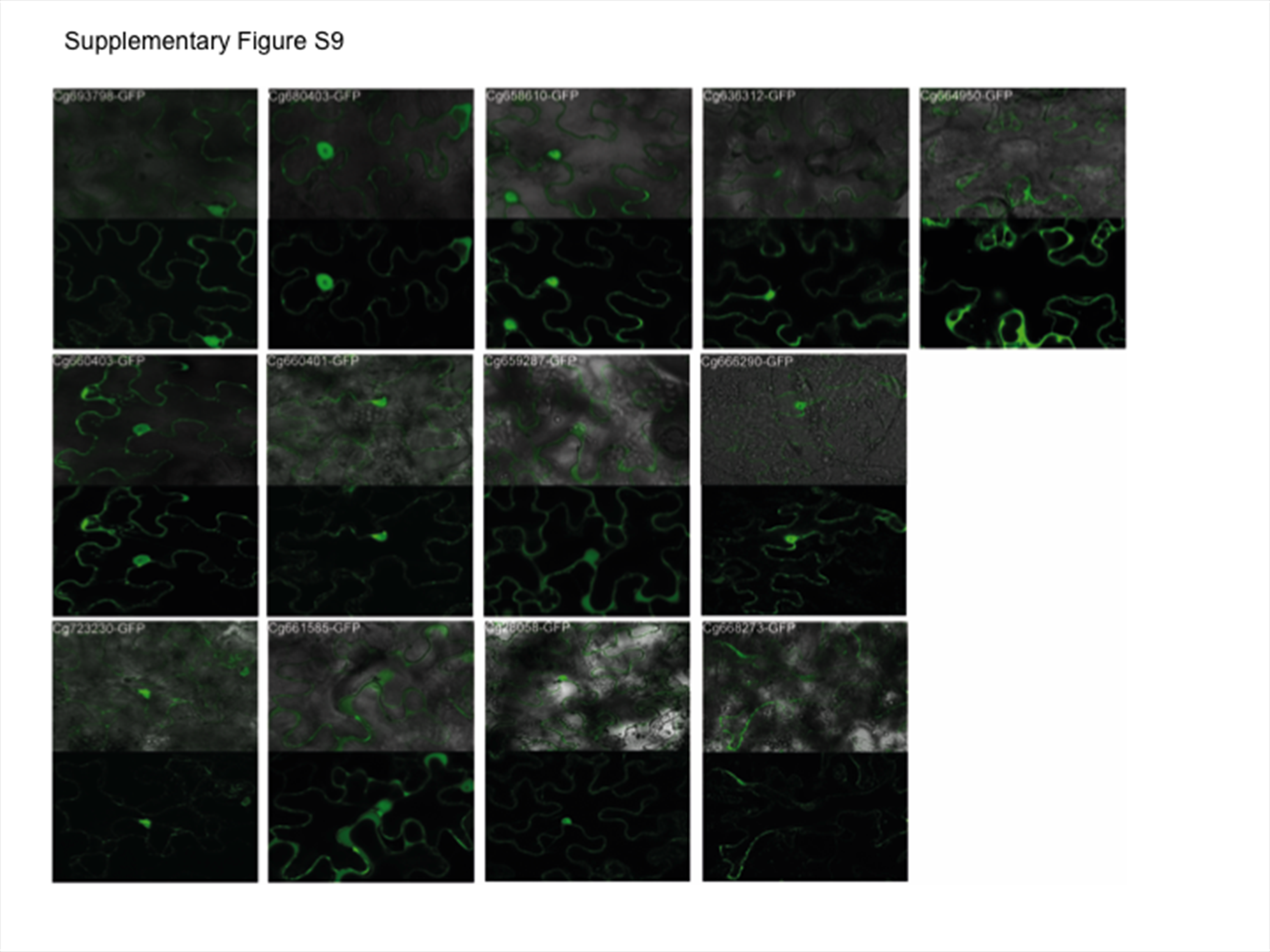

Supplement: Supplementary Figure S9 — Candidate effectors with no informative localization in planta. Representative images corresponding to the 13 fusion proteins accumulating in the nucleoplasm and the cytosol. The fusion proteins were transiently expressed in Nicotiana benthamiana leaf cells by agroinfiltration. Live-cell imaging was performed with a laser-scanning confocal microscope 2 days after infiltration. The green fluorescent protein (GFP) was excited at 488 nm. GFP (green) fluorescence was collected at 505–525 nm. [file Image9.TIFF]

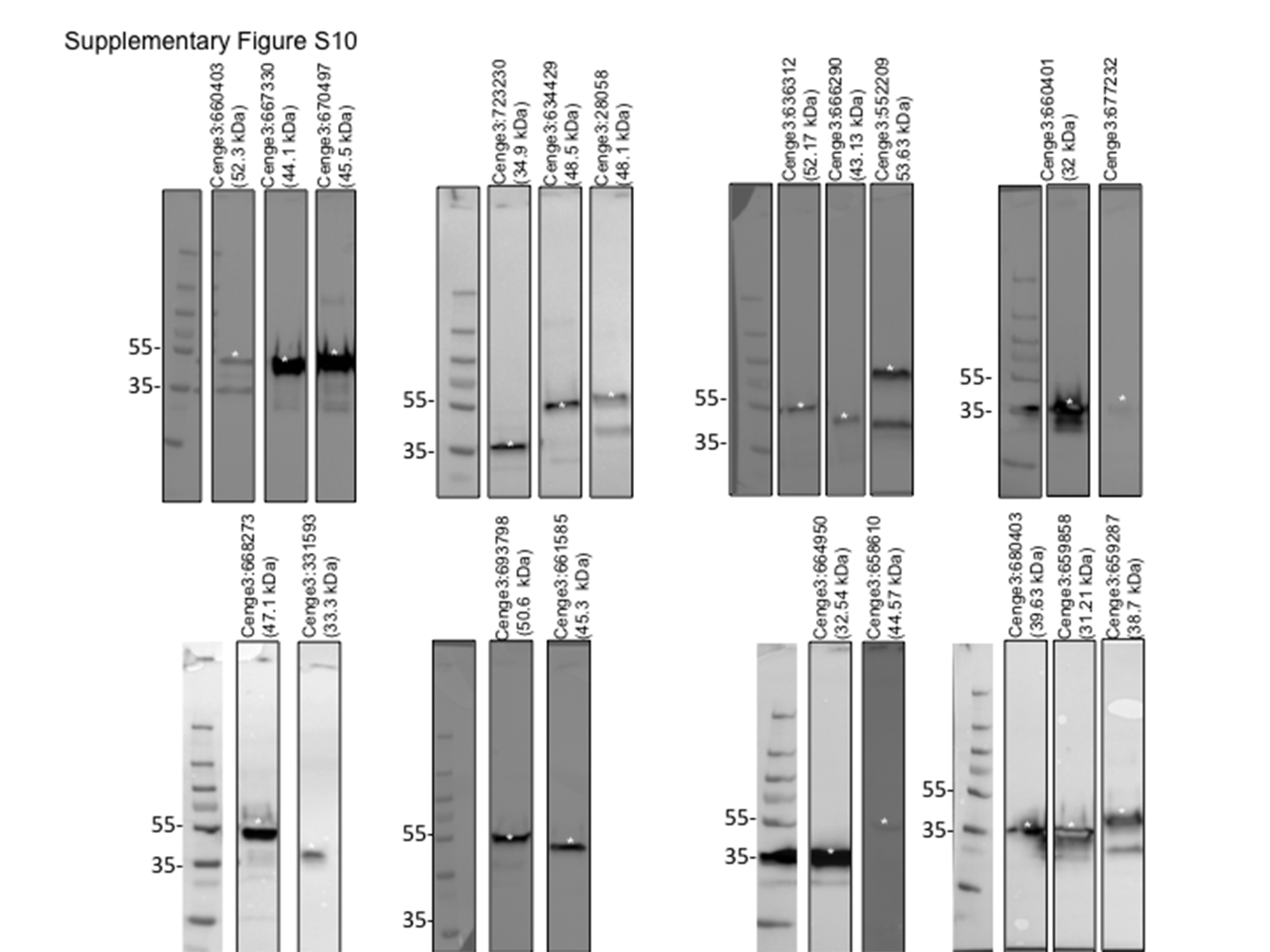

Supplement: Supplementary Figure S10 — Immunoblots of CgMiSSPs:GFP fusion proteins in N. benthamiana leaves. GFP detection was performed in a single step by a GFP-HRP conjugated antibody. The theoretical size of each fusion protein (SSP+GFP) is indicated between parentheses in kiloDalton (kDa). Page rulers and corresponding sizes in kiloDalton (kDa) are indicated on the blots. White asterisks indicate specific protein bands. [file Image10.TIFF]
