# Supplementary material for: Secretome Analysis from the Ectomycorrhizal Ascomycete Cenococcum geophilum
Source: Front Microbiol. 2018 Feb 13;9:141. doi: 10.3389/fmicb.2018.00141 (PMC5816826; doi:10.3389/fmicb.2018.00141)
Supplement: Supplementary Figure S3 — Nucleotide (A–C) and protein (D–F) alignments of duplications of candidate MiSSPs in the C. geophilum genome. (A,D) Cenge3:636312 and Cenge3:660403, (B,E) Cenge3:679266 and Cenge3:693798, (C,F) Cenge3:660401 and Cenge3:659858. Protein ID from Joint Genome Institute (JGI). [file Image3.PDF]

Supplementary Figure S3

A

```
          1        10        20        30        40        50        60        70        80        90        100       110       120      130
636312    ATGAAGTTCAGCACACTTTCAGTCGGCCTCCTCGGCCTTGTCTCCTCCGCCTACTCCGCTGCCATCGAACCCGCCGTGGTCAAACGTGACACTAAACTCCGCTTTGAAGTCCAATTCAGCGACAAACTTG
660403    ATGAAGTTCAGCATACTTTCACTCGGCCTCCTTGGCCTTGTCTCCTTCGCCTACTCCGCCGCCATCAAACCCGCCGTGGTTAAACGTGACACTAAACTCCACTTTGAAGTCCAATTCAGCGACAAACTTA
Consensus ATGAAGTTCAGCAcACTTTCAcTCGGCCTCCTcGGCCTTGTCTCCTcCGCCTACTCCGCcGCCATCaAACCCGCCGTGGTcAAACGTGACACTAAACTCCaCTTTGAAGTCCAATTCAGCGACAAACTTa

          131      140       150       160       170       180       190       200       210       220       230       240       250      260
636312    TCGACGTTGGTAATCTCAACTCCTTCGATGCCGTCTGGCAGCGCATGTACGCTCAATCCGACGACCATGCTGGTCTCTCTGATGCTACCTACTATCAATTCGACGGCGCCTGCAACCACGAACTCGAGAA
660403    TCGACGTTGGTAATCTTAACTCCTTCGATGCCGTCTGGCAGCGCATGTACGCTCAATCCAACGATCATGCTGGTCTCTCTGATGCTACCTACTATCAATTCGACAGCGCCTGCAACCACAAATTCGACAA
Consensus TCGACGTTGGTAATCTcAACTCCTTCGATGCCGTCTGGCAGCGCATGTACGCTCAATCCaACGAcCATGCTGGTCTCTCTGATGCTACCTACTATCAATTCGACaGCGCCTGCAACCACaAAcTCGAcAA

          261      270       280       290       300       310       320       330       340       350       360       370       380      390
636312    GCCGAAGCTTAACGTCGATGTTAAGCTTGACGGTCAATGGGGCAACTTTGGCGATTTCAATAATTGGGACATGCGCAACTCTGTCATCAAAACTATGTGGGCTGCGGTCGACAACCTGGGCAAACAGCAA
660403    GCCTCAGCTTAACGTTAATGTTAAGCTTGACGGTCAATGGGGCAACTTTGGCGATTTTAATAACTGGGACATGCGCAACGCTGTTATTAAAACTATGTGGGCTGCGGTTGATAACTTAGGTAAACAGCAA
Consensus GCCgaAGCTTAACGTcaATGTTAAGCTTGACGGTCAATGGGGCAACTTTGGCGATTTcAATAAcTGGGACATGCGCAACgCTGTcATcAAAACTATGTGGGCTGCGGTcGAcAACcTaGGcAAACAGCAA

          391      400       410       420       430       440       450       460       470       480       490       500       510      520
636312    AGCCGCTCGATCTTCAGCAAATGCCGTGGCACCACTTGGCAGGAATCAGTGGCGTACTCGGAAAATGCAGCCTGCGGTCCTTTAGTTGCAATCAAGGACTGCCAGTGCGGTATCTATACCGCGCAGTGTA
660403    AGCCGCTTAATCTTCAGTAAATGCTATGGCACCGTTTGGCAGGAAATAGTGGCGTACACGGAAAATGCAGCCTGCGGTCGTTTGGCTGCAATTAAGGACTGCCAGTGCGATATCCTTGCCGCGCAGTGTA
Consensus AGCCGCTcaATCTTCAGcAAATGCcaTGGCACCacTTGGCAGGAAacAGTGGCGTACaCGGAAAATGCAGCCTGCGGTCcTTTaGcTGCAATcAAGGACTGCCAGTGCGaTATCcaTaCCGCGCAGTGTA

          521      530       540       550       560       570       580       590       600       610       620       630       640      650
636312    TACAAGTGGACAATGGCTTCATGCTACCATCCGTCATCAAGGTGCTTGTGTATCAGAACGATGGTGTGCTTCGCCCAGATAACTACATCGTCACTTTGTCGTCCTACAAGGCTACAAGCGCAGGTGGCTG
660403    TGCAAGTGGACAATGGCTTCATGCTACCATCTATTATTAAGGTGCTTGTGTATCAGAGCGATGGTGTGCTTCGCCCAGATAACTATATCGTTACTTTATCGTCCCACAAGGCTACAAGCCAAGGTGGTTG
Consensus TaCAAGTGGACAATGGCTTCATGCTACCATCcaTcATcAAGGTGCTTGTGTATCAGAaCGATGGTGTGCTTCGCCCAGATAACTAcATCGTcACTTTaTCGTCCcACAAGGCTACAAGCcaAGGTGGcTG

          651      660       670       680       690       700       710       720       730       740    747
636312    TGGAAAGGCTGGTGCGCTTGGTGAAGTCCTTGCCAGCTTTATTCCGGGTATTGGTACCTTCATCGAGAAGGGTGTCAAGGTTGCTTGCCGTCAGTAA
660403    TGGAAAGGCTGGTGCGCTTGCTGAAGTCCTTGCTAGCTTTATCCCGGGTATTGGTACCTTCGTTGAGAAGGGTATTAAGGTTACTTGCCGTCAATAA
Consensus TGGAAAGGCTGGTGCGCTTGcTGAAGTCCTTGCcAGCTTTATcCCGGGTATTGGTACCTTCaTcGAGAAGGGTaTcAAGGTTaCTTGCCGTCAaTAA
```

B

```
          1        10        20        30        40        50        60        70        80        90        100       110       120      130
660401    ATGATTCTCCCTCGATTCTTCGCAATCTTCTTTGCCGCCATGTCTATGGTGCAAGCTCAGGCAATGCCTAAGCTTGACACTCGCGAGCTTCAAGTTCGCAATGATGGTACTGCGTGCCCAGAGGCTGGTG
659858    ATGATTCTCCCTCGATTCTTCGCAATTTTCTTTGTCGCCATGTCTATGGTGCAAGCTCAGGCGATGCCTGAGCTTGACACTCGCGAGCTTCAAGTTCGCGATGATGGTACTGCGTGCCCAGAGGCTGGTG
Consensus ATGATTCTCCCTCGATTCTTCGCAATcTTCTTTGcCGCCATGTCTATGGTGCAAGCTCAGGCaATGCCTaAGCTTGACACTCGCGAGCTTCAAGTTCGCaATGATGGTACTGCGTGCCCAGAGGCTGGTG

          131      140       150       160       170 174
660401    AAGGCCCTTACGATCCAGCTCTGTATTTCGACCACCAGTGCTAG
659858    AGGGCCCTCACGATCCAGCTCTGTATTTCGACCACCAGTGCTAG
Consensus AaGGCCCTcACGATCCAGCTCTGTATTTCGACCACCAGTGCTAG
```

C

```
          1        10        20        30        40        50        60        70        80        90        100       110       120      130
679266    ATGCGCTTTACACTCTTAACGTTCTCAGCACTTATTCTCAT---ATGGCACCAAGCTCTTGCTTTACCAGGCGCGTTGCCTCCCACACCTAATACAGAAGTAACTGTCTGGAATCTCCCTCGCACGGCTC
693798    ATGCGCTTCACGCTTCCAACGCTCTCGGCACTCATCCTCCTCACATGGCACCAGGCTCTTGCTTTGCCAGGCATGTCGCCCTCTACACCCAATACGAAAGAAGCTGTCTGGAATCTCCCTCGCGCGGCTC
Consensus ATGCGCTTcACaCTcccAACGcTCTCaGCACTcATcCTCaT...ATGGCACCAaGCTCTTGCTTTaCCAGGCacGTcGCCccCcACACCcAATACaaAAGaAaCTGTCTGGAATCTCCCTCGCaCGGCTC

          131      140       150       160       170       180       190       200       210       220       230       240       250      260
679266    CATATGACGCCGCCACGCACCTAATGTTAGACCCAAGAGCCGTTAATGTTAAGGCCACTGCCAACATTGACACTCGCGGCCTTCAAGCTCGTGCCCTACCCTGGTCATCATCTGAATTAAAGCGCCGGTT
693798    TATATGACGCCGCCACGCACCAAATGTTAGACTCGAGGGCCGTTGATGTTGAGGCCACTGACGACCTCGACACCCGCAGCCTTCAAACCCGCGCCATTCCCTGGCAATCAGCTGACTTGAAGCGCCGGTT
Consensus cATATGACGCCGCCACGCACCaAATGTTAGACcCaAGaGCCGTTaATGTTaAGGCCACTGaCaACaTcGACACcCGCaGCCTTCAAaCcCGcGCCaTaCCCTGGcaATCAgCTGAaTTaAAGCGCCGGTT

          261      270       280       290       300       310       320       330       340       350       360       370       380      390
679266    GGTTGTCGGGCTGTCGACCGCTGTCGCCACTACGATCCATCTCGCCAACGCCGCCATAACCTGGAAACTCGCCTTAGTATATCACACGGATGGCAATGGCATTGAGACAGTGACAGGGAATGTCTACCCA
693798    GGTCGTCGGGCTGTCGACCGCCGTCGCCACTTCGATCCATCTCGCCAACGCCGCGCTGACCTGGAAATTCTCCTTAGTATATCACACGGATTTGAGTGGCCGCGAGACATTAACATCGACACTCTACGAA
Consensus GGTcGTCGGGCTGTCGACCGCcGTCGCCACTaCGATCCATCTCGCCAACGCCGCcaTaACCTGGAAAcTCgCCTTAGTATATCACACGGATggcAaTGGCagcGAGACAgTaACAgcGAacTCTACcaA

          391      400       410       420       430       440       450       460       470       480       490       500       510      520
679266    GATTGA
693798    GATGGAGAAACGAAGTTACAGAACGTGAATCTGTGTGTAAAAGGGTTTACAAGTTCTGTATCTGGCAACACCGCTAATGGCTATTGGGTGGACGCGGATGTCGATTTGGAGGGCAGCTACGTTGATACTG
Consensus GATgGA............................................................................................................................

          521      530       540       550       560       570       580       590       600       610       620       630       640      650
679266
693798    CCTCGGGGAATGCGGTTACGATGTTTTGGACAGCGGCCATGCGTTTGGGCCAGTATGCCGCGTCAAACTATGTCCAACATATGGTACACGATCCCACAGGATATGAGCGGATTCCGGAGGACACTTCACA
Consensus ..................................................................................................................................

          651      660       670       680       690       700       710    717
679266
693798    GATGTGCCTAATCCCGGGTGGCACGTCGGTTAATCCTACTTCCTGGACATTTGATGCATGGACTTGA
Consensus ...................................................................
```

D

```
           1        10        20        30        40        50        60        70        80        90        100       110       120       130
           |--------+---------+---------+---------+---------+---------+---------+---------+---------+---------+---------+---------+---------|
   636312  MKFSTLSVGLLGLVSSAYSAAIEPAVVKRDTKLRFEVQFSDKLVDVGNLNSFDAVWQRMYAQSDDHAGLSDATYYQFDGACNHELEKPKLNVDVKLDGQWGNFGDFNNWDMRNSVIKTMWAAVDNLGKQQ
   660403  MKFSILSLGLLGLVSFAYSAAIKPAVVKRDTKLHFEVQFSDKLIDVGNLNSFDAVWQRMYAQSNDHAGLSDATYYQFDSACNHKFDKPQLNVNVKLDGQWGNFGDFNNWDMRNAVIKTMWAAVDNLGKQQ
Consensus  MKFSiLSlGLLGLVSfAYSAAIePAVVKRDTKLrFEVQFSDKL!DVGNLNSFDAVWQRMYAQS#DHAGLSDATYYQFDgACNHel#KPqLNV#VKLDGQWGNFGDFNNWDMRNaVIKTMWAAVDNLGKQQ

           131      140       150       160       170       180       190       200       210       220       230       240     248
           |--------+---------+---------+---------+---------+---------+---------+---------+---------+---------+---------+-------|
   636312  SRSIFSKCRGTTWQESVAYSENAACGPLVAIKDCQCGIYTAQCIQVDNGFMLPSVIKVLVYQNDGVLRPDNYIVTLSSYKATSAGGCGKAGALGEVLASFIPGIGTFIEKGVKVACRQ
   660403  SRLIFSKCYGTVWQEIVAYTENAACGRLAAIKDCQCDILAAQCMQVDNGFMLPSIIKVLVYQSDGVLRPDNYIVTLSSHKATSQGGCGKAGALAEVLASFIPGIGTFVEKGIKVTCRQ
Consensus  SRlIFSKCrGTtWQEiVAYsENAACGrLaAIKDCQCdIlaAQCiQVDNGFMLPS!IKVLVYQnDGVLRPDNYIVTLSShKATSaGGCGKAGALaEVLASFIPGIGTF!EKG!KVaCRQ
```

E

```
           1        10        20        30        40        50     57
           |--------+---------+---------+---------+---------+------|
   659858  MILPRFFAIFFVAMSMVQAQAMPELDTRELQVRDDGTACPEAGEGPHDPALYFDHQC
   660401  MILPRFFAIFFAAMSMVQAQAMPKLDTRELQVRNDGTACPEAGEGPYDPALYFDHQC
Consensus  MILPRFFAIFFaAMSMVQAQAMPeLDTRELQVR#DGTACPEAGEGPhDPALYFDHQC
```

F

```
           1        10        20        30        40        50        60        70        80        90        100       110       120       130
           |--------+---------+---------+---------+---------+---------+---------+---------+---------+---------+---------+---------+---------|
   679266  MRFTLLTFSALILI-WHQALALPGALPPTPNTEVTVWNLPRTAPYDAATHLMLDPRAVNVKATANIDTRGLQARALPWSSSELKRRLVVGLSTAVATTIHLANAAITWKLALVYHTDGNGIETVTGNVYP
   693798  MRFTLPTLSALILLTWHQALALPGMSPSTPNTKEAVWNLPRAALYDAATHQMLDSRAVDVEATDDLDTRSLQTRAIPWQSADLKRRLVVGLSTAVATSIHLANAALTWKFSLVYHTDLSGRETLTSTLYE
Consensus  MRFTLlTlSALILi.WHQALALPGalPpTPNTeeaVWNLPRaAlYDAATHqMLDpRAV#VeATa#iDTRgLQaRAiPWqSa#LKRRLVVGLSTAVATsIHLANAAiTWKlaLVYHTDgnGrETlTgnlYe

           131      140       150       160       170       180       190       200       210       220       230     238
           |--------+---------+---------+---------+---------+---------+---------+---------+---------+---------+-------|
   679266  D
   693798  DGETKLQNVNLCVKGFTSSVSGNTANGYWVDADVDLEGSYVDTASGNAVTMFWTAAMRLGQYAASNYVQHMVHDPTGYERIPEDTSQMCLIPGGTSVNPTSWTFDAWT
Consensus  D...........................................................................................................
```
